# Interpretable ensemble learning model using intratumoral and peritumoral multi-sequence MR-radiomics predicts high-grade cervical cancer with lymphovascular space invasion

**DOI:** 10.3389/fonc.2026.1827751

**Published:** 2026-09-09

**Authors:** Fei Wang, Chunyue Yan, Jiqiang He, Xiaolin Tang, Kun Wang, Deqiang Xian

**Affiliations:** 1 Department of Radiology, Luzhou People’s Hospital, Luzhou, China; 2 Department of Emergency Medicine, Luzhou People’s Hospital, Luzhou, China; 3 Department of Pathology, Luzhou People’s Hospital, Luzhou, China; 4 Department of Administrative Office, Luzhou People’s Hospital, Luzhou, China

**Keywords:** cervical cancer, lymphovascular space invasion, machine learning, MRI, radiomics

## Abstract

**Objective:**

To develop an interpretable ensemble learning model for predicting dual positivity of pathological grading and lymphovascular space invasion (HGVI) in cervical cancer (CC) by integrating multiple machine learning algorithms, fusion strategies, and multi-sequence MR radiomics.

**Methods:**

A total of 242 CC patients who underwent preoperative MRI were retrospectively enrolled and randomly divided into a training set (n = 169) and a test set (n = 73). Volumes of interest were manually delineated on intratumoral and peritumoral (3 mm and 5 mm) regions across three MR sequences of apparent diffusion coefficient (ADC), T1-weighted imaging (T_1_WI) and T2-weighted imaging (T_2_WI). Six machine learning classifiers were employed, and the optimal base models were subsequently integrated via mean, weighted averaging, and logistic regression-based stacking (ENMLR). Model performance was evaluated using the area under the curve (AUC), confidence intervals (CI), and decision curve analysis (DCA). Interpretability was examined through SHAP, correlation, and restricted cubic spline (RCS) analyses.

**Results:**

Compared with the eight models, the ENMLR achieved superior performance in the training set, which was robustly validated in the test set via 1,000 Bootstrap resamples (AUC: 0.858, 95% CI: 0.762–0.932). In the test set, the ENMLR demonstrated good accuracy (0.7260), a Brier score of 0.1523, and enhanced clinical net benefit across a wide threshold range (10–90%). Correlation analyses revealed positive agreement between intratumoral and peritumoral radiomics (Spearman’s r: 0.15, 0.21, 0.41, and 0.29; all P < 0.05). RCS analysis further identified a significant nonlinear relationship between ADC sequence-derived intratumoral radiomics scores and an increased incidence of HGVI double positivity (Non-linear P < 0.05; Overall P < 0.05), with a notable inflection point. SHAP analysis identified ADC_peritumoral_3mm and the Xgboost classifier as the top contributors to the fusion model.

**Conclusions:**

By integrating diverse learning algorithms with multi-sequence MR radiomics (intratumoral and peritumoral) and serum biomarkers, the ENMLR demonstrates robust discriminative performance for predicting HGVI double positivity in CC. Nonetheless, further multicenter prospective studies are warranted to validate these findings.

## 1 Introduction

Cervical cancer (CC) is a significant global health issue and exhibits high heterogeneity. Due to the high recurrence rate and metastasis rate of CC, there were approximately 341,831 global deaths from cervical cancer and 604,127 new cases reported in 2020 (1). Additionally, the National Comprehensive Cancer Network (NCCN) guideline has indicated that adjuvant radiotherapy is necessary for CC patients with confirmed high-risk pathological feature risk factors, such as lymphovascular space invasion (LVSI), degree of pathological differentiation and lymph node metastasis, following radical hysterectomy for CC (2). LVSI refers to the spread of cancer cells within lymphatic and/or microvessels, and is a very important independent prognostic factor for evaluating the prognosis of CC (3–6). The degree of pathological differentiation is also crucial for evaluating the preoperative treatment decisions of CC patients (7–11). However, the histopathological evaluation of CC remains invasive, complex, and inherently limited. Moreover, most recent studies have predominantly focused on a single pathological endpoint (3–11), lacking a comprehensive and synchronized analysis of multiple pathological features. Therefore, non-invasive preoperative synchronous evaluation of multiple high-risk pathological features such as double positivity of High pathological grading and LVSI (HGVI) is crucial for optimizing treatment decisions and stratified management of CC patients.

Radiomics, an innovative image analysis method, can reflect the internal heterogeneity and microenvironmental changes of tumors (12). Previous studies have found that functional parameters of MRI, such as apparent dispersion coefficient (ADC) value, k value, f and D* value, are regarded as important tools for non-invasive preoperative assessment of the pathological grade of CC (7–9, 11). However, different radiologists have some subjective factors and limitations in region of interest (ROI) measurement for CC, and only perform ROI annotation at the maximum level and lacking of a standardized ROI measurement method. Furthermore, the above ROI annotation is two-dimensional rather than three-dimensional, lacking a comprehensive evaluation of the heterogeneity of the entire tumor microenvironment, including necrosis, inflammatory response, and infiltration of small lymphatic vessels and microvessels within the overall lesion of CC; Importantly, the microenvironment infiltration of lymphatic vessels and microvessels around the tumor has not been quantified. Moreover, most previous studies only evaluated a single pathological feature and did not comprehensively analyze pathological grading and LVSI together (3–11). Yang T et al. (4) included 155 patients with CC to explore the efficacy of radiomics and deep learning in preoperative prediction of LVI. In the validation cohort, the AUC value of the deep learning model was 0.745, and that of the radiomics model was 0.686. Furthermore, Liu Y et al. (10) included 160 CC patients to explore the differences between the 2D radiomics characteristics and the 3D radiomics characteristics of the total tumor volume in the pathological grading of CC. The results showed that the 3D radiomics analysis was superior to the 2D central sections of the tumor in the histological grading of CC. However, all the above-mentioned studies are based on the intratumor of CC patients, but lack the quantification and study of the peritumor microenvironment of CC.

From a biological standpoint, the complementary value of intratumoral and peritumoral regions can be explained by their distinct pathophysiological implications (13). Intratumoral radiomics features primarily characterize the internal heterogeneity of CC, such as necrosis, cellular density, and vascular irregularity, which are closely associated with tumor grade and aggressiveness (8, 14). In contrast, peritumoral radiomics features capture subtle changes in the adjacent stroma, including inflammatory cell infiltration, fibroblastic activation, and early lymphovascular invasion (15). Thus, the MR-based radiomics for predicting high-risk pathologic features (High pathological grading and lymphovascular space invasion) still has some challenges. focusing only on a single high-risk pathological feature may overlook the significant impact on the treatment decisions of CC patients when multiple high-risk pathological features coexist. It is not known whether multi-phase stacking MR-radiomics has a beneficial effect for predicting HGVI double positivity in CC patients. Moreover, different machine learning algorithms, including k-nearest neighbors (KNN), logistic regression (LR), extreme gradient boosting classifiers (Xgboost), support vector machines (SVM), Bayes, and artificial neural networks (NNet), exhibit distinct characteristics and have been widely applied in cancer research.

Therefore, this study aims to compare and develop an interpretable ensemble learning model for predicting HGVI double positivity using different machine learning algorithms, multi-sequence MRI radiomics, and intratumoral and peritumoral radiomics and serum biomarkers. Moreover, different machine learning fusion strategies were evaluated, and the clinical utility and interpretability of the models were investigated.

## 2 Materials and methods

### 2.1 Patients

This retrospective study was approved by the Ethics Committee of our institution (Approval No. LLW202501022). Written informed consent was waived.

All patients with CC histologically were diagnosed from July 2016 to August 2025. Inclusion criteria included: (a) Age ≥ 18 years, and the pathological grades and LVSI were clear. According to the WHO Classification of Tumors of Female Reproductive Organs (16, 17), all CC patients of pathological grades were divided into two groups: High-grade (moderately differentiated or poorly differentiated CC) and Low-grade (well differentiated CC). Moreover, the LVSI of all patients was clear and classified as LVSI-positive and LVSI-negative; (b) The MRI examination was completed within four weeks before surgery, including three sequences of apparent diffusion coefficient (ADC), T1-weighted imaging (T_1_WI), and T2-weighted imaging (T_2_WI); and (c) Complete serum biomarkers examination within two weeks before surgery. Exclusion criteria included: (a) The image quality was poor; (b) Presence of a history of preoperative anti-tumor treatment; (c) Presence of other concurrent malignant tumors or autoimmune diseases.

Additionally, the HGVI double positivity of CC was defined both High-grade and LVI-positive, while in the case of Non-dual-positive group of HGVI, three subgroups were defined: 1) High-grade and LVSI-negative, 2) Low-grade and LVSI-positive and 3) Low-grade and LVSI-negative. **Supplementary Figure 1** shows the flowchart of patient screening. Ultimately, 242 CC patients were analyzed in this study.

### 2.2 Baseline information and MR scanning techniques

Patient demographic and clinical laboratory data, including age, white blood cell count, neutrophil count, alanine aminotransferase (ALT), alkaline phosphatase (ALP), gamma-glutamyl transferase (GGT), carcinoembryonic antigen (CEA), Carbohydrate antigen 125 (CA125), etc, were collected. Optimal cut-off values for predicting HGVI dual-positivity of CC were determined through receiver operating characteristic curve (ROC) analysis (the maximize Youden index [sensitivity+specificity−1]) (18–20). **Supplementary Table 1** summarizes the optimal cutoff values. Feature selection was performed using the Boruta algorithm and the least absolute shrinkage and selection operator (LASSO) regression to identify optimal clinical biomarkers, and the logistic regression score of clinical biomarkers (CLS) was constructed by using their common subset.

All MR imaging was performed on a 3.0 T scanner (Siemens Skray). The protocol included three sequences: ADC (b=800 s/mm^2^), T_1_WI, and T_2_WI. Detailed scanning parameters were provided in **Supplementary Table 2**.

### 2.3 Image preprocessing and tumor segmentation

Firstly, perform N4 bias field correction on all MR images. secondly, AII MR images were resampled to 1mm × 1mm × 1mm to minimize scanner variability effects by linear interpolation to standardize voxel spacing. Subsequently, the MR images of the three sequences were registered, with the T_2_WI sequence as the reference, and the T_1_WI and ADC images were rigidly registered. Finally, All MR Images underwent intensity normalization and gray-level discretization (bin width=25) for standardization.

Tumor segmentation was conducted using 3D Slicer (version 5.0.2, https://www.slicer.org/). Two radiologists (Reader 1 and Reader 2, each with 11 years of experience in MRI interpretation) manually delineated the tumors on preprocessed MR images to create three-dimensional volumes of interest (VOIs). Peritumoral regions extending 3 mm and 5 mm from the tumor boundary were automatically generated via the “Margin” module in 3D Slicer, followed by removal of blood vessels and adjacent tissues. To evaluate feature consistency, a subset of 50 randomly selected CC patients underwent repeat segmentation independently by Reader 1 and Reader 3 (With 25 years of clinical working experience) after a one-month interval. The intraclass correlation coefficient (ICC) was used to assess the repeatability and stability of the radiomic features.

### 2.4 Radiomics feature extraction and selection

Radiomic features were extracted using the PyRadiomics package. A total of 10,170 features (1130 × 9) were derived from intratumoral, peritumoral 3 mm, and peritumoral 5 mm regions across ADC, T_1_WI, and T_2_WI sequences. Importantly, all the dimensionality reduction processes of the radiomics features and the training models of the machine learning algorithms were carried out independently within the training set and were invisible to the test set.

After normalization, only features with an intraclass correlation coefficient (ICC) > 0.80 were retained. Dimensionality reduction was performed through the following steps: (a) screening for group differences using t-tests or Mann–Whitney U tests (P ≤ 0.05); (b) removing highly correlated features (Spearman’s correlation > 0.9); (c) applying Maximum Relevance Minimum Redundancy (mRMR) selection; (d) retaining the top ten most contributory features via the SHapley Additive exPlanations (SHAP) algorithm. The Boruta algorithm was then used to identify the most important features for modeling. Based on the non-zero coefficients and weights of these selected features, radiomics scores were calculated for each region (intratumoral, peritumoral 3 mm, peritumoral 5 mm) and sequence (ADC, T_1_WI, T_2_WI). Finally, logistic regression was applied to construct stacked radiomics scores for intratumoral (intraStacking) and peritumoral (periStacking) regions from three sequences.

### 2.5 Model building and evaluation

In the training set, the univariate and multivariate logistic regression were used to screen the predictors. The odds ratio (OR) becomes an evaluation indicator of independent risk predictors. Overall, independent factors that had a P<0.05 were utilized for the subsequent development of model. Ultimately, six machine-learning models of Bayes, LR, KNN, Xgboost, SVM, and NNet were built in the training set. Importantly, several fusion strategies of the optimal model was integrated for all machine learning prediction probability using mean (ENMM), weighted average (ENMWR), and logistic regression (ENMLR).

The model’s performance was evaluated using the area under the curve (AUC), accuracy, precision, recall, F1 score, and Brier score. The calibration curve and decision curve analysis (DCA) were utilized to assess the model’s calibration capability and net benefit. The imbalanced data of the categories were analyzed using the balanced accuracy and the precision-recall AUC (PR-AUC).

### 2.6 Model interpretability

**Figure 1:**
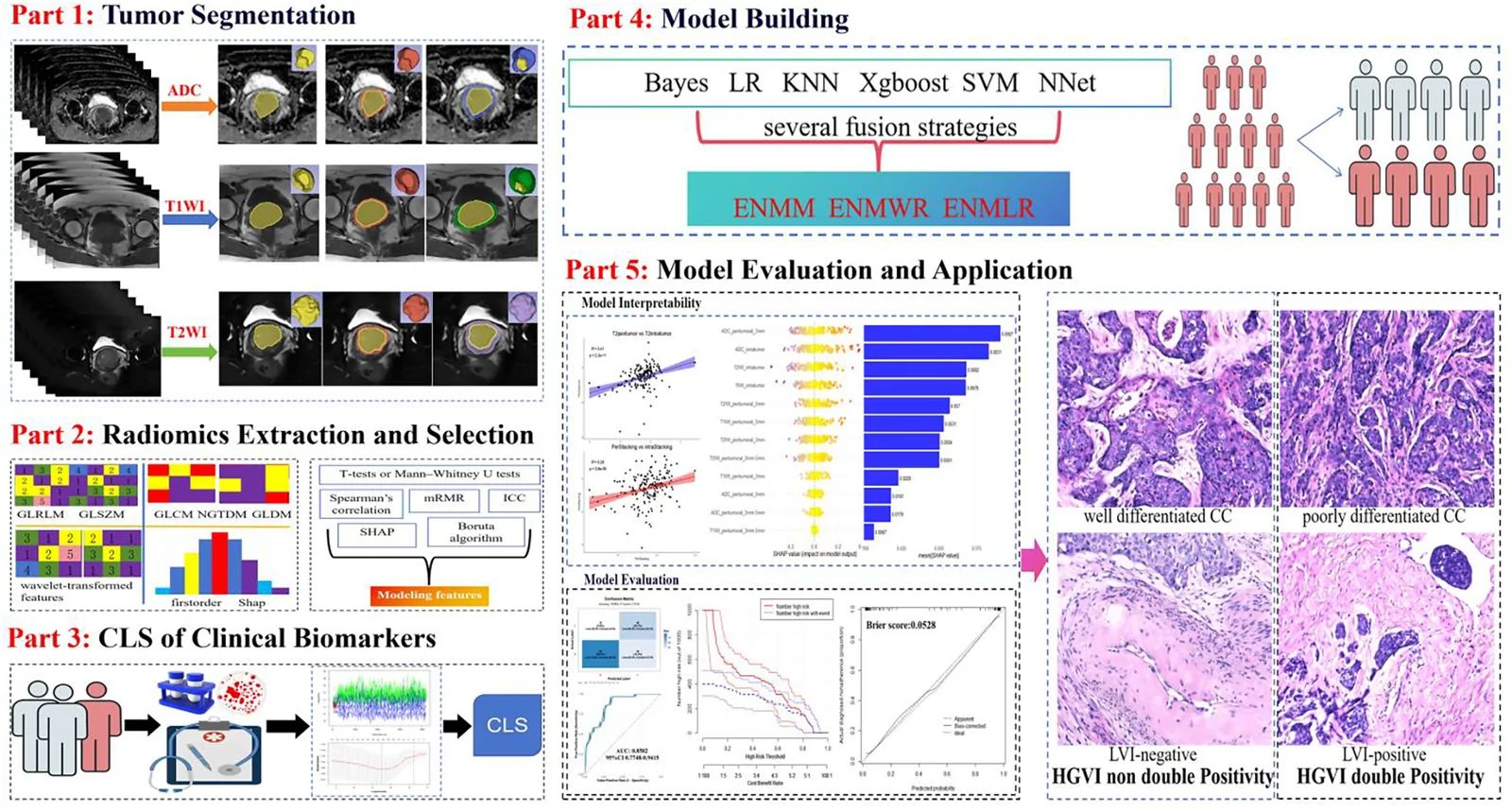
The design and workflow of this study.

To assess the association between intratumoral and peritumoral predictions, Pearson and Spearman correlation analyses were conducted on the respective predicted probabilities. Restricted cubic spline (RCS) analysis was further performed to evaluate concordance between intratumoral and peritumoral radiomics scores. Model interpretability was examined using the SHapley Additive exPlanations (SHAP) framework, which generated global feature importance rankings and SHAP force plots to delineate the contributions of individual features, thereby facilitating the biological and clinical interpretation of the radiomics signatures. **Figure 1** summarizes the overall workflow of this study.

### 2.7 Statistical analysis

All analyses were performed using R 4.1.2 (*http://www.r-project.org/**, Austria*) and MedCalc (*Version 15.2*). Classifiers were developed in R utilizing multiple machine learning packages (mlr3, glm, rms, glmnet, caret, kNN, e1071, Xgboost). Continuous variables are presented as mean ± SD when normally distributed (independent-sample t-test) or analyzed with Mann-Whitney U test otherwise. Categorical variables were compared using Pearson χ2 test or Fisher exact probability test. Statistical significance was defined as two-tailed P < 0.05.

## 3 Results

### 3.1 Baseline characteristics

Ultimately, a total of 242 patients with CC were included in this study and randomly allocated to a training set (n=169) and a test set (n=73) in a 7:3 ratio. The rates of HGVI double positivity were 37.87% (64/169) in the training set and 31.51% (23/73) in the test set, with no statistically significant difference between the two sets (P = 0.423). Baseline characteristics showed no significant differences in training set except for neutrophil count (P = 0.043), and no significant differences were observed in test set (all P<0.05). These baseline characteristics are summarized in **Table 1**. Additionally, the optimal cut-off values for predicting HGVI double positivity of serum biomarkers in both sets are provided in **Supplementary Table 3**.

**Table 1:** The baseline characteristics of HGVI double positivity and non-double positivity in CC patients.

Variables	Training set			Test set		
	Non-dual-positive group(N = 105)	Dual-positive group(N = 64)	P values	Non-dual-positive group(N = 50)	Dual-positive group(N = 23)	P values
Age	61.3 (11.2)	59.3 (10.3)	0.224	59.2 (12.9)	61.1 (9.8)	0.483
WBC(×10^9^/L)	6.68 (2.3)	8.99 (11.1)	0.104	7.58 (2.6)	7.13 (2.5)	0.476
Neu(×10^9^/L)	4.64 (2.1)	5.80 (4.2)	0.043	6.05 (5.6)	5.20 (2.6)	0.385
Lym(×10^9^/L)	1.51 (0.6)	1.74 (2.5)	0.460	2.37 (5.8)	1.50 (0.5)	0.295
MO(×10^9^/L)	0.45 (0.2)	1.32 (6.2)	0.269	0.72 (1.5)	0.42 (0.2)	0.170
PLT(×10^9^/L)	259 (90.6)	253 (91.8)	0.682	258 (64.2)	245 (104.0)	0.589
ALT(IU/L)	19.8 (13.7)	20.2 (24.7)	0.903	14.8 (7.9)	19.2 (10.4)	0.083
AST(IU/L)	23.0 (11.4)	25.0 (21.1)	0.493	19.5 (6.2)	22.8 (10.3)	0.166
ALP(IU/L)	92.3 (29.9)	93.1 (34.0)	0.880	86.2 (22.6)	96.0 (43.8)	0.321
GGT(IU/L)	30.2 (30.4)	28.7 (25.6)	0.723	22.9 (15.9)	27.4 (26.3)	0.460
CA199(U/mL)	27.6 (69.2)	54.9 (77.0)	0.241	31.6 (83.7)	30.7 (37.1)	0.949
CA125(U/mL)	38.3 (84.7)	81.2 (192.0)	0.095	34.6 (68.9)	137 (269.0)	0.085
CEA(U/mL)	6.72 (10.5)	12.2 (41.5)	0.300	5.47 (5.3)	21.7 (60.4)	0.212

WBC, white blood cell count; Neu, neutrophil count; Lym, lymphocyte count; MO, monocyte count; PLT, platelet count; ALT, alanine aminotransferase; AST, aspartate aminotransferase; ALP, alkaline phosphatase; GGT, Gamma-glutamyl transferase; CA199, Carbohydrate antigen 199; CA125, Carbohydrate antigen 125; CEA, Carcinoembryonic antigen; HGVI, High pathological grading and lymphovascular space invasion; CC, Cervical cancer.

### 3.2 Optimal feature selection and stacking

**Figure 2:**
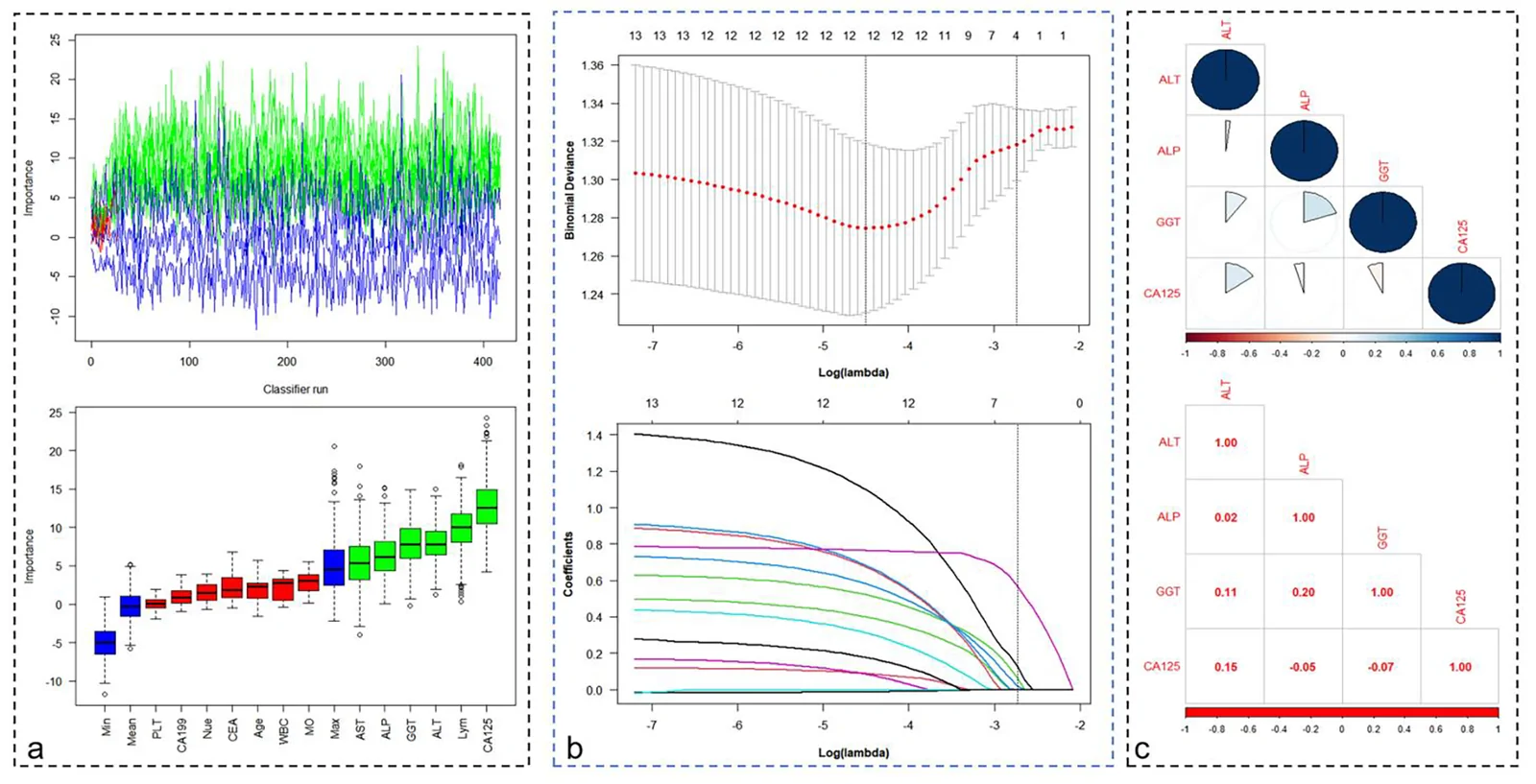
Features screening of clinical baseline characteristics using Boruta algorithm and LASSO. When classifier run 400 times, the importance of confirmed feature was obviously higher than those of shadow features and tentative features, and six features were included **(a)**; The selection path plots of LASSO regression **(b)** and tenfold cross-validation, and four features were included **(b)**; The heat map analysis of the four most important features **(c)**. LASSO, least absolute shrinkage and selection operator.

For 13 clinical biomarkers, the Boruta algorithm was used to screen and underwent 400 iterations (**Figure 2a**), with 6 feature selections. Similarly, the LASSO regression was used to screen and performed five fold cross validation (**Figure 2b**), with 4 features selected. Ultimately, the common subset of the two methods is the four important features (**Supplementary Table 4**). Heat map analysis indicates that the correlation among the four important features is relatively weak (**Figure 2c**). Ultimately, the CLS for predicting HGVI double positive in CC is as follows:

CLS==-2.122206 + 1.139281*ALT
+0.7832072*ALP
+0.5940868*GGT
+1.250619*CA125

For radiomics, the radiomic features with poor reproducibility and ICC<0.8 were eliminated. After a series of feature dimensionality reduction, the mRMR was gradually used to screen for the features with the highest redundancy. The number of feature filtering process is summarized in **Supplementary Table 5**. Furthermore, **Supplementary Figure 2** summarizes the detailed radiomics features screening of peritumoral 3 mm, 5 mm and intratumor based on SHAP algorithm and Boruta algorithm. Ultimately, the intraStacking was calculated based on intratumoral radiomics features of ADC, T_1_WI and T_2_WI, and the perStacking was calculated based on peritumoral 3mm and peritumoral 5mm radiomics features of ADC, T_1_WI and T_2_WI. **Supplementary Table 6** provides a detailed processes of the intraStacking, and perStacking. The performance of intraStacking and perStacking for preoperative prediction of HGVI double positive in CC is summarized in **Table 2**. **Supplementary Figure 3** suggests that the peritumoral radiomics has supplementary value for intratumoral radiomics in predicting HGVI double positive in both the training set (AUC: increased from 0.694 to 0.774) and the test set (AUC: increased from 0.709 to 0.775).

**Table 2:** The performance of intraStacking and perStacking for predicting HGVI double positive of CC in training set and test set.

Variables	Training set			Test set		
	AUC	SE	95%CI	AUC	SE	95%CI
perADCRS	0.649	0.0447	0.572 to 0.721	0.626	0.0692	0.505 to 0.737
perT1WIRS	0.616	0.0447	0.538 to 0.690	0.674	0.0662	0.554 to 0.779
perT2WIRS	0.680	0.0414	0.604 to 0.750	0.679	0.0649	0.559 to 0.783
perStacking	0.733	0.0406	0.660 to 0.798	0.712	0.0648	0.595 to 0.812
ADCRS	0.530	0.0498	0.452 to 0.607	0.614	0.074	0.492 to 0.725
T1WIRS	0.630	0.0435	0.552 to 0.703	0.613	0.0666	0.492 to 0.725
T2WIRS	0.649	0.0423	0.572 to 0.721	0.629	0.0658	0.507 to 0.739
intraStacking	0.694	0.0407	0.619 to 0.763	0.709	0.0617	0.591 to 0.810
perStacking+intraStacking	0.774	0.0373	0.704 to 0.835	0.775	0.0585	0.662 to 0.865

AUC, area under the curve; CI, confidence interval; SE, standard error; ADC, apparent diffusion coefficient; T1WI, T1-weighted imaging; T2WI, T2-weighted imaging; RS, the radiomics score of radiomics features; perStacking, the stacking radiomics score based on peritumoral 3mm and peritumoral 5mm radiomics features of ADC, T1WI and T2WI; intraStacking, the stacking radiomics score based on intratumoral radiomics features of ADC, T1WI and T2WI; HGVI, High pathological grading and lymphovascular space invasion; CC, Cervical cancer.

### 3.3 Independent predictors for HGVI double positivity

**Supplementary Figure 4** provides a detailed demonstration of independent predictors screening process using the univariate and multivariate logistic regression analysis. Eventually, the results indicated that perStacking (OR = 2.102[95%CI 1.329-3.326]; P = 0.002), intraStacking (OR = 2.389[95%CI 1.394-4.094]; P = 0.002), and CLS (OR = 2.968[95%CI 1.737-5.075]; P<0.001) were independent predictors for predicting HGVI double positivity of CC.

### 3.4 Model building and model fusion

Six machine learning models were established to evaluate performance for predicting HGVI double positivity of CC based on the perStacking, intraStacking and CLS. **Supplementary Figure 5** summarizes ROC curves and confusion matrices of six machine learning. Importantly, compared with the five machine learning models, Xgboost achieved higher AUC (0.9685 *vs.* 0.8268), higher F1 score (0.8594 *vs.* 0.7000), lower Brier score (0.1044 *vs.* 0.1703) on the training set and test set.

**Figure 3:**
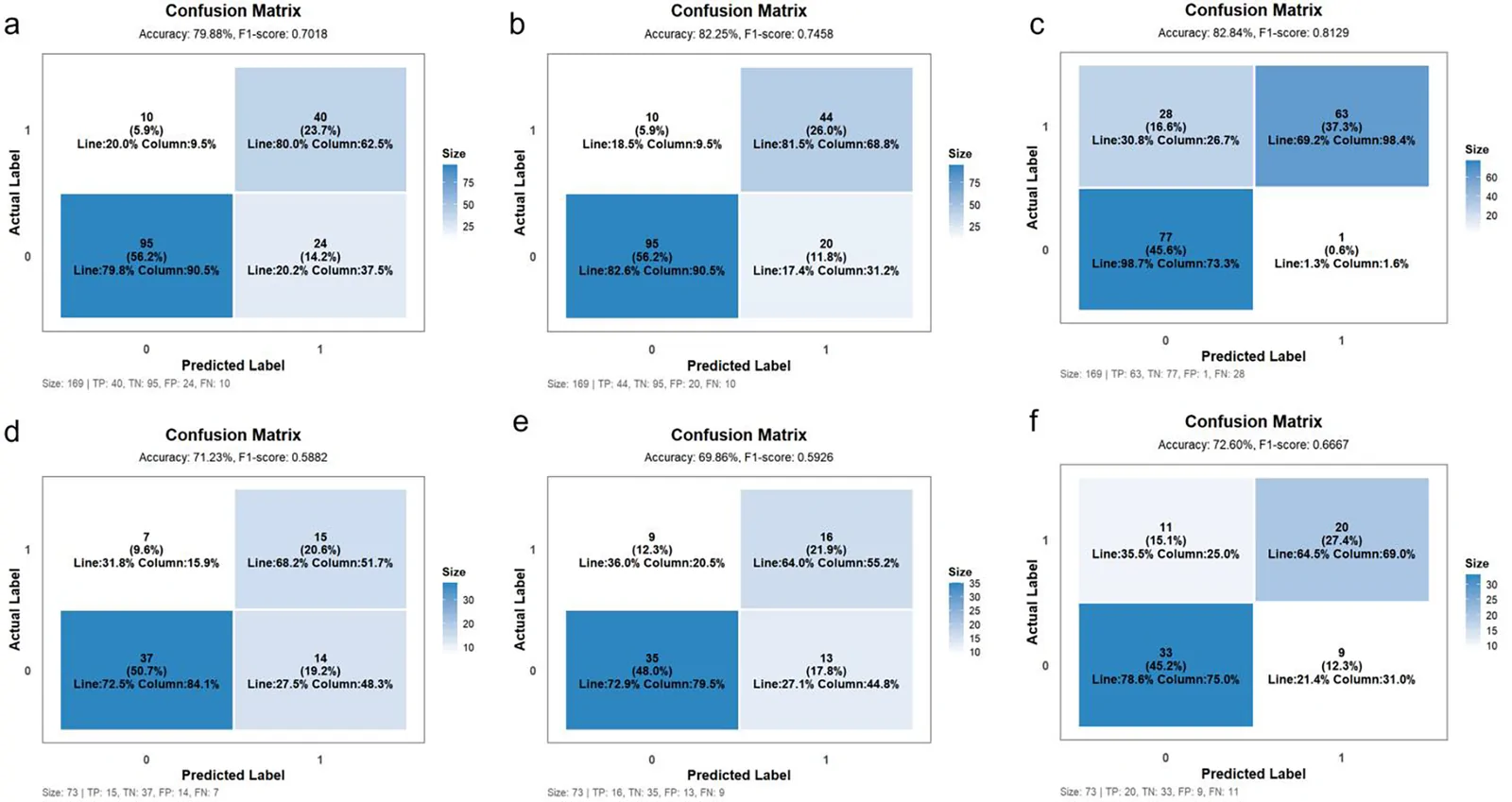
Confusion matrix of ENMM **(a, d)**, ENMWM **(b, e)**, and ENMLR **(c, f)** for predicting HGVI double positivity of CC in training set **(a-c)** and test set **(d-f)**. ENMM, the ensemble model was integrated for all machine learning prediction probability using mean; ENMWM, the ensemble model was integrated for all machine learning prediction probability using weighted average; ENMLR, the ensemble model was integrated for all machine learning prediction probability using logistic regression; HGVI, High pathological grading and lymphovascular space invasion; CC, Cervical cancer.

Moreover, based on the prediction probabilities of all machine learning models, several fusion strategies of the ENMM, ENMWR, and ENMLR were integrated by using mean, weighted average, and logistic regression. **Figure 3** summarizes confusion matrices of three fusion models.

The 9 models for predicting HGVI double positivity are summarized in **Table 3**. To be more specific, the ROC curve analysis indicated that ENMLR obtained more predictive performance (**Supplementary Figure 6**) in the training set and test set, and DCA also indicated that ENMLR achieved more clinical net benefits to predict HGVI double positivity of CC than the other models (**Supplementary Figure 7**).

**Table 3:** The performance of 9 models for predicting HGVI double positivity of CC in training set and test set.

Models	AUC(95%CI)	Accuracy	Precision	Recall	F1-score	Brier-score
Training set						
Bayes	0.8186(0.7551-0.8821)	0.7515	0.6562	0.6774	0.6667	0.1687
KNN	0.8294(0.7646-0.8942)	0.7811	0.5938	0.7755	0.6726	0.1546
LR	0.8253(0.7630-0.8876)	0.7337	0.6667	0.5938	0.6281	0.1629
NNet	0.8975(0.8514-0.9435)	0.8343	0.6562	0.8750	0.7500	0.1193
SVM	0.8304(0.7600-0.9007)	0.7929	0.5938	0.8085	0.6847	0.1531
Xgboost	0.9685(0.9471-0.9898)	0.8935	0.8594	0.8594	0.8594	0.1044
ENMM	0.9012 (0.8564-0.9460)	0.7988	0.6250	0.8000	0.7018	0.1319
ENMWM	0.9301 (0.8945-0.9656)	0.8225	0.6875	0.8148	0.7458	0.1202
ENMLR	0.9787 (0.9615-0.9959)	0.8284	0.9844	0.6923	0.8129	0.0528
Test set						
Bayes	0.7531 (0.6362-0.8701)	0.7260	0.6207	0.6667	0.6429	0.1987
KNN	0.7206 (0.5991-0.8421)	0.6986	0.4828	0.6667	0.5600	0.2110
LR	0.7571(0.6413-0.8728)	0.7260	0.6957	0.5517	0.6154	0.1917
NNet	0.7155(0.5953-0.8357)	0.6849	0.4828	0.6364	0.5490	0.2426
SVM	0.7461(0.6307-0.8615)	0.6986	0.4483	0.6842	0.5417	0.2004
Xgboost	0.8268(0.7333-0.9203)	0.7534	0.6774	0.7241	0.7000	0.1703
ENMM	0.7688 (0.6584-0.8792)	0.7123	0.5172	0.6818	0.5882	0.1889
ENMWM	0.7821 (0.6769-0.8873)	0.6986	0.5517	0.6400	0.5926	0.1866
ENMLR	0.8582 (0.7748-0.9415)	0.7260	0.6897	0.6452	0.6667	0.1523

KNN, k-nearest neighbor; LR, logistic regression; NNet, neural network; SVM, support vector machine; Xgboost, extreme gradient boosting classifiers; ENMM, the ensemble model was integrated for all machine learning prediction probability using mean; ENMWM, the ensemble model was integrated for all machine learning prediction probability using weighted average; ENMLR, the ensemble model was integrated for all machine learning prediction probability using logistic regression; AUC, area under the curve; CI, confidence interval; HGVI, High pathological grading and lymphovascular space invasion; CC, Cervical cancer.

### 3.5 Model evaluation and 1,000 bootstrap resamples

**Figure 4:**
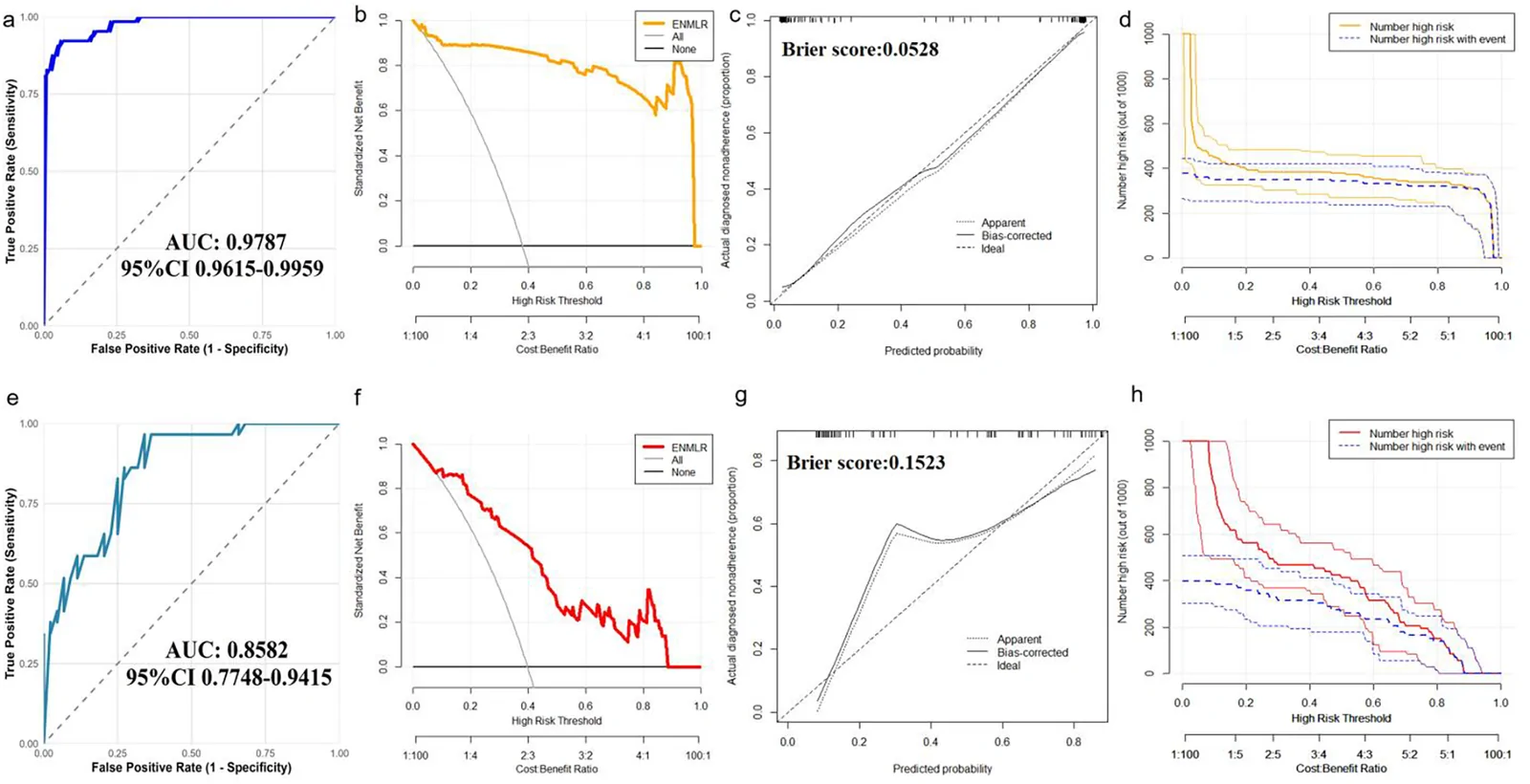
Verification and evaluation of the ENMLR. The ROC curve **(a, e)**, DCA curve **(b, f)**, calibration curve **(c, g)** and clinical impact curve **(d, h)** of the ENMLR for predicting HGVI double positivity of CC in training set **(a-d)**, and test set **(e-h)**. ROC, receiver operating characteristic curve; ENMLR, the ensemble model was integrated for all machine learning prediction probability using logistic regression; HGVI, High pathological grading and lymphovascular space invasion; CC, cervical cancer.

**Figure 4** presents the evaluation and validation of the ENMLR. In the test set, at a cutoff of 0.5, the model exhibited an accuracy of 0.7260, a precision of 0.6897, an F1 score of 0.6667, and a recall of 0.6452. The DCA indicated that the ENMLR yielded greater clinical net benefits across threshold probabilities ranging from 10% to 90%. Calibration curves demonstrated good consistency, with Brier scores of 0.0528 and 0.1523 for the training and test sets, respectively. The Hosmer–Lemeshow goodness-of-fit test further confirmed no significant difference between the predicted and observed values, supporting adequate model calibration in both the training (χ2 = 15.061, P = 0.058) and test (χ2 = 14.468, P = 0.070) sets. Additionally, the CIC suggested that the ENMLR could deliver substantial net benefits across a wide threshold range, indicating that this model is worthy of promotion for routine clinical practice.

**Figure 5:**
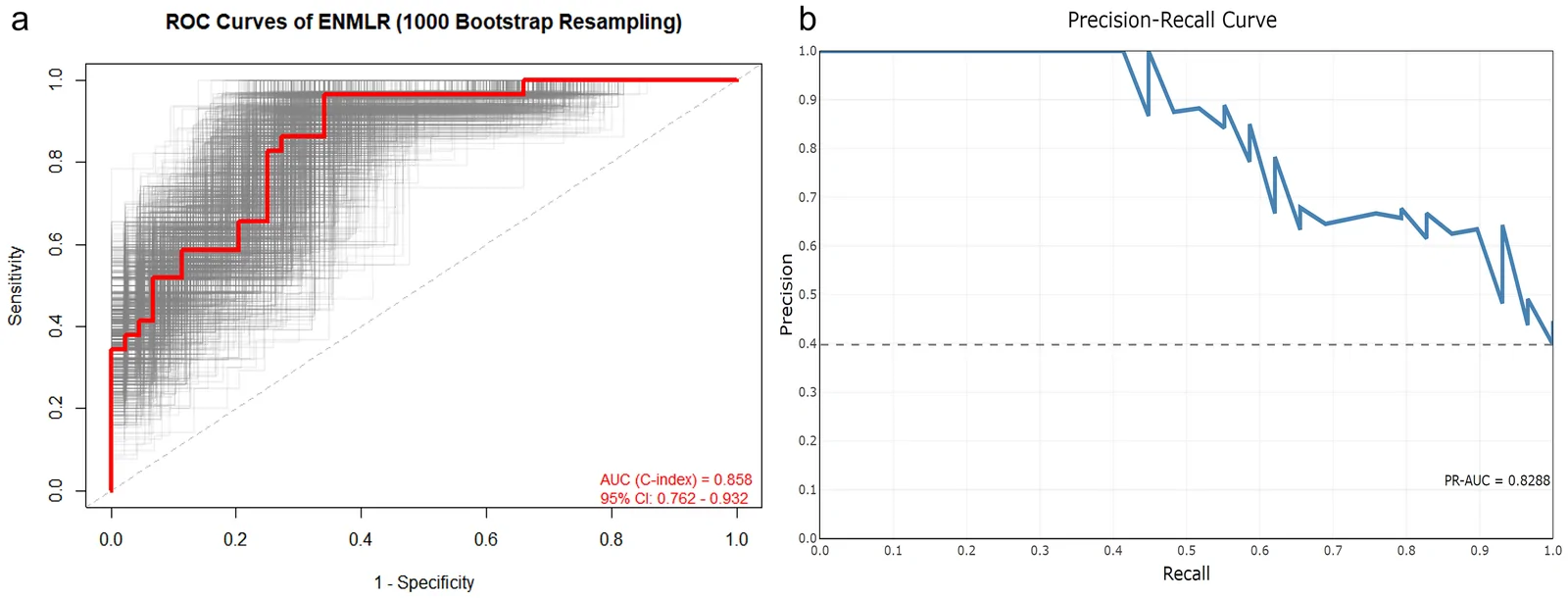
Bootstrap 1,000 resamples **(a)** and the precision-recall ROC curve analysis **(b)** in the test set. For the ENMLR, this 1,000 bootstrap resampling approach yielded an AUC of 0.858 (95% CI: 0.762–0.932) on the test set **(a)**. Given the inherent class imbalance in our data—37.87% in the training set and 31.51% in the test set, and the ENMLR achieved a balanced accuracy of 0.7198 and a PR-AUC of 0.8288 in the test set. ROC, receiver operating characteristic curve; ENMLR, the ensemble model was integrated for all machine learning prediction probability using logistic regression; AUC, area under the curve; CI, confidence interval; CC, cervical cancer.

To assess internal validity, 1,000 bootstrap samples (with replacement) were drawn from the training set, each subjected to the complete feature selection workflow of the original pipeline. For the ENMLR, this 1,000 bootstrap resampling approach yielded an AUC of 0.858 (95% CI: 0.762–0.932) on the test set (**Figure 5a**). Given the inherent class imbalance in our data—37.87% (64/169) in the training set and 31.51% (23/73) in the test set—we prioritized balanced accuracy and the precision-recall AUC (PR-AUC) alongside the conventional ROC-AUC (**Figure 5b**). The ENMLR achieved a balanced accuracy of 0.7198 and a PR-AUC of 0.8288 in the test set, affirming robust discriminative performance across both classes and underscoring the clinical relevance of the model.

### 3.6 Model interpretability analysis

**Figure 6:**
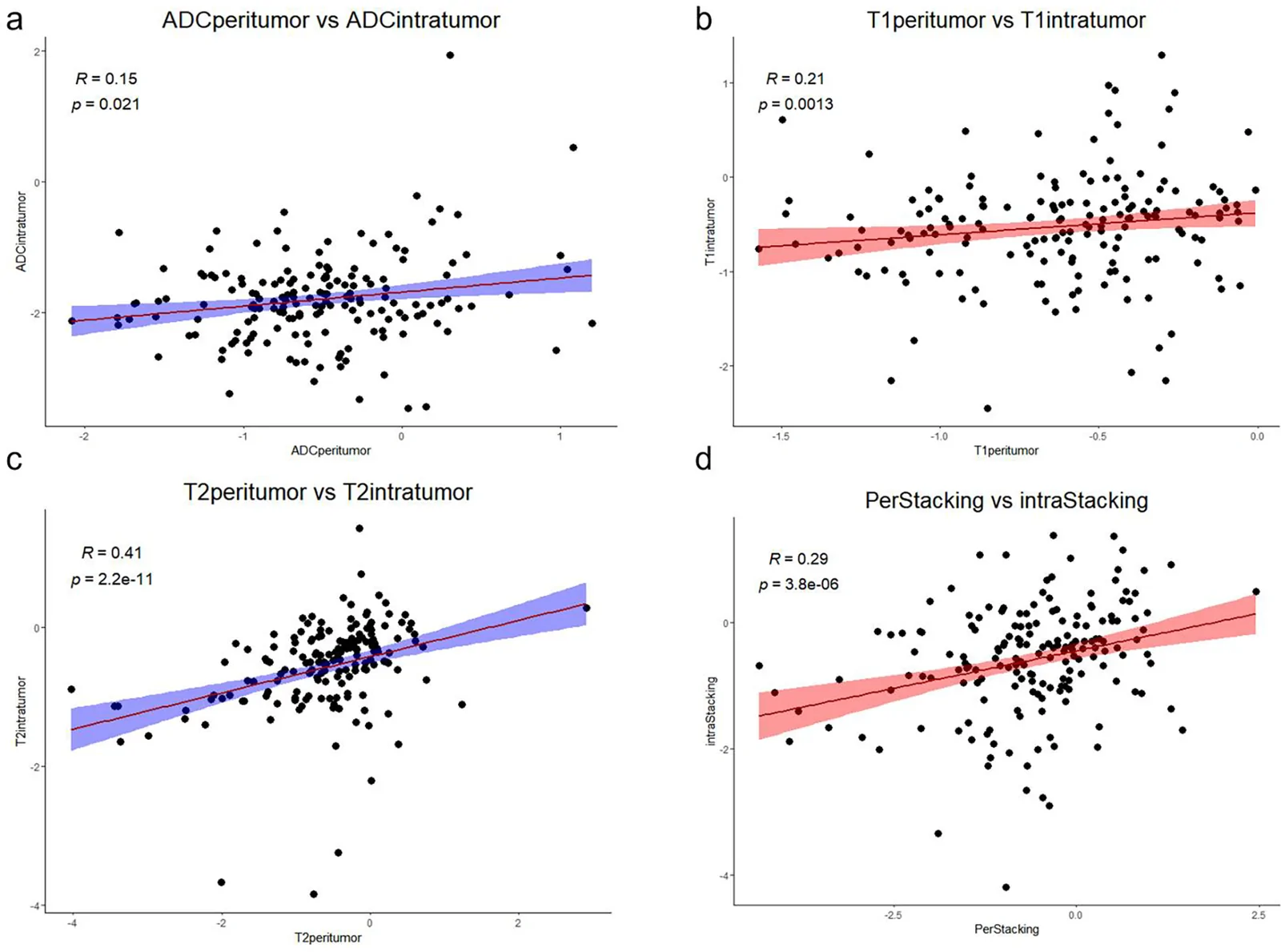
Correlation analysis for the peritumoral and intratumoral radiomics features. The scatter plot suggests a significant positive correlation between the peritumoral and intratumoral radiomics features in the ADC sequence **(a)**, T_1_WI sequence **(b)**, T_2_WI sequence **(c)**, and stacking radiomics **(d)**. ADC, apparent diffusion coefficient; T_1_WI, T1-weighted imaging; T_2_WI, T2-weighted imaging; HGVI, High pathological grading and lymphovascular space invasion; CC, cervical cancer.

As shown in **Figure 6**, the peritumoral and intratumoral radiomics features of the ADC sequence, T_1_WI sequence, T_2_WI sequence, and stacking radiomics have a certain positive correlation (Spearman’s r: 0.15, 0.21, 0.41, 0.29; all P < 0.05), indicating that the peritumoral and intratumoral radiomics features have a synergistic effect in quantifying the pathological grade and lymphovascular space invasion of CC.

**Figure 7:**
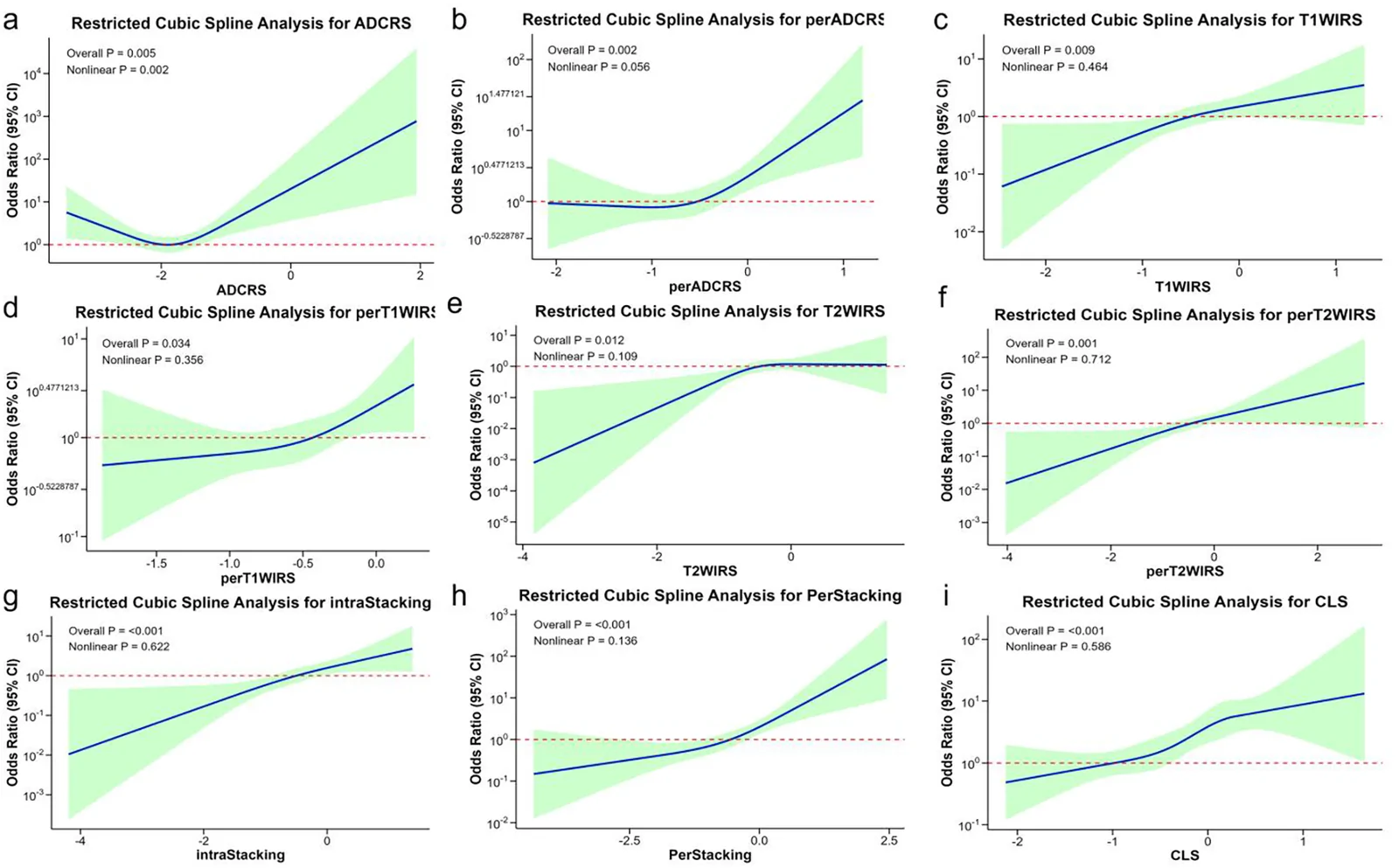
Restrictive cubic spline analysis for the peritumoral and intratumoral radiomics features of ADC sequence **(a, b)**, T_1_WI sequence **(c, d)**, T_2_WI sequence **(e, f)**, stacking radiomics **(g, h)**, and CLS **(i)**. The results suggest that the intratumoral radiomics scores of ADC sequence exhibits significant nonlinear relationships for predicting HGVI double positive of CC (Nonlinear P<0.05; Overall P<0.05), with the intratumoral radiomics features of ADC sequence demonstrating significant inflection point effects; However, no nonlinear relationship was observed for the other features. ADC, apparent diffusion coefficient; T_1_WI, T1-weighted imaging; T_2_WI, T2-weighted imaging; RS, the radiomics score of radiomics features; perStacking, the stacking radiomics score based on peritumoral 3mm and peritumoral 5mm radiomics features of ADC, T_1_WI and T_2_WI; intraStacking, the stacking radiomics score based on intratumoral radiomics features of ADC, T_1_WI and T_2_WI; HGVI, High pathological grading and lymphovascular space invasion; CC, Cervical cancer.

As shown in **Figure 7**, the RCS analysis showed a significant nonlinear correlation (Nonlinear P<0.05; Overall P<0.05) between the intratumoral radiomics scores of ADC sequence and the increased incidence of HGVI double positive of CC; Importantly, the intratumoral radiomics scores of ADC sequence demonstrates significant inflection point effects. However, no nonlinear relationship was observed for the other features. When these features (except for the ADC sequence) are at lower levels, it is associated with the decreased incidence of HGVI double positivity in CC. However, as these indicator values increase, the ratio also gradually rises.

**Figure 8:**
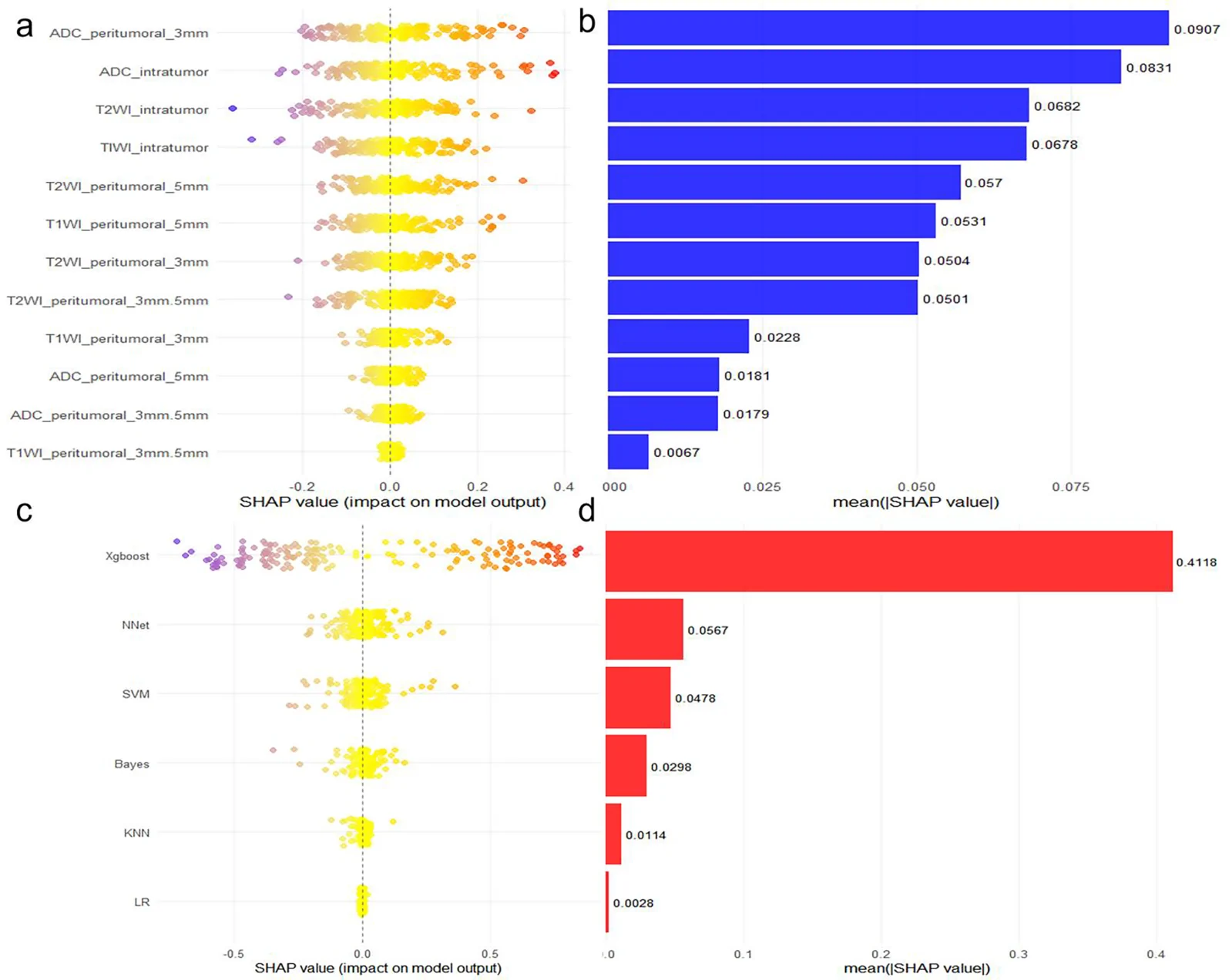
SHAP analysis of the model. The result suggests that the ADC_peritumoral_3mm contributed the most to the model, with an average Shapley value of 0.0907 **(a, b)**. The machine learning of Xgboost achieved the greatest contribution, with an average Shapley value of 0.4118 **(c, d)**. SHAP, the SHapley Additive exPlanations; ADC, apparent diffusion coefficient; T_1_WI, T1-weighted imaging; T_2_WI, T2-weighted imaging; HGVI, High pathological grading and lymphovascular space invasion; CC, cervical cancer.

As shown in **Figure 8**, the SHAP analysis could enhance the interpretation and visualization of the importance of each feature in the optimal model. For the peritumoral and intratumoral radiomics features of the ADC sequence, T_1_WI sequence, and T_2_WI sequence, the ADC_peritumoral_3mm contributed the most for the fusion model, achieving a Shapley value mean of 0.0907. Notably, in the final fusion model, the machine learning of Xgboost made the greatest contribution, with a Shapley value mean of 0.4118.

## 4 Discussion

To comprehensively and simultaneously evaluate the high-risk pathological features (pathological grading and lymphovascular space invasion) in CC patients, we innovatively developed a hybrid model that integrates six distinct machine learning algorithms and three fusion strategy, multi-sequence MRI radiomics (including intratumoral and peritumoral regions), and serum biomarkers. The ENMLR was robustly validated in the test set via 1,000 Bootstrap resamples (AUC: 0.858, 95% CI: 0.762–0.932) for predicting HG-MVI double positivity of CC, along with an accuracy of 0.726, a Brier score of 0.152, and greater clinical net benefit across a wide threshold probability range (10–90%). This hybrid model exhibited optimal predictive performance, discriminative ability and stability, may offer potential for guiding personalized surgical decisions and stratified management of high-risk CC patients.

From a biological perspective, the intratumoral radiomics features reflect the internal heterogeneity of CC, including necrosis, cell density, and vascular irregularities, which correlate with tumor pathological grade and invasiveness (8, 14). Similarly, peritumoral radiomics features capture subtle alterations in the adjacent stroma, such as inflammatory cell infiltration, fibroblast activation, and early lymphovascular invasion (15). Several recent studies have explored the roles of multi-sequence MR radiomics in assessing LVSI in CC (4, 21, 22). For instance, Liu FH et al. (21) included 177 CC patients, with results showing that the AUCs of radiomics nomogram and mpMRI models were 0.838 and 0.835 in the training set, and 0.837 and 0.817 in the test set, respectively. Additionally, Yang T et al. (4) included 155 CC patients, with the AUC of the radiomics model (Rad) being 0.801 and the AUC in the validation set being 0.686. Similarly, Deng L et al. (22) included 196 CC patients, demonstrating that the AdaBoost-based mpMRI model achieved an AUC of 0.953 in the training set, 0.868 in internal validation, and 0.797 in external validation. However, all the above-mentioned multi-sequence MR radiomics were solely used to evaluate the LVSI of CC, lacking analysis of peritumoral 3mm and 5mm radiomics for the CC patients, nor did they simultaneously assess the high-risk pathological features of pathological grade and LVSI for CC patients. From a clinical perspective, accurately preoperatively assessing HGVI dual positivity for CC patients is crucial for determining surgical strategies, including the extent of lymphadenectomy and the selection of neoadjuvant therapy. In this study, peritumoral radiomics significantly provided supplementary value in preoperatively predicting HGVI dual positivity, indicating that peritumoral 3 mm and 5 mm radiomics of the CC patients captured peritumoral microstructural and microenvironmental characteristics not reflected by traditional clinical variables. Therefore, the stacking of these two imaging domains provides a more comprehensive tumor biological description for the synchronous assessment of high-risk pathological features of CC.

Theoretically, the intratumoral radiomics focuses on the imaging features within a tumor, such as texture, shape and intensity, which reflect the heterogeneity and biological behavior of the tumor (23). The peritumoral radiomics can reflect the invasive ability and micrometastases (24). Importantly, compared with the well differentiated tumors, the poorly differentiated tumors have higher heterogeneity, with higher entropy values, gray level co-occurrence matrix parameters and histogram features (25). In this study, it was worth noting that peritumoral 3mm and 5mm radiomics from ADC, T_1_WI and T_2_WI were independent predictive biomarkers for predicting HGVI double positivity in CC, and most of the features come from the wavelet features of the firstorder, glszm, gldm, original_shape_Elongation and SmallAreaLowGrayLevelEmphasis. These features effectively reveal the biological characteristics of the non-uniformity, heterogeneity, necrosis and microangiogenesis of tumor cell proliferation (26); Similarly, the degree of pathological grading reflects the spatial heterogeneity and invasiveness of intratumor for CC patients, while LVSI reflects the intratumor and surrounding microenvironment dominated by microvascular and lymphatic infiltration. Thus, the HGVI double positivity of CC often exhibit pronounced peritumoral infiltration, inflammatory responses, microtransfer, and angiogenesis, with more aggressive. Our findings were similar to those of previous studies that radiomics features such as wavelet features, texture (e.g., gray-level inhomogeneity of the peritumor region), and morphological parameters (e.g., peritumoral infiltration extent) correlated significantly with tumor differentiation (24, 25). Importantly, this study has proved that combining intratumoral and peritumoral MR radiomics features enhances model accuracy and robustness, providing a comprehensive assessment of CC biological behavior and high-risk pathological features.

In addition to differentiating manifestations, correlation and interpretability analysis also provides a deeper insight into the different but complementary roles of the regions within and around the tumor. SHAP analysis showed that the peritumor feature of ADC_peritumoral_3mm contributed more to the final joint prediction, which implies that the peri-tumor region representing the tumor microenvironment plays a key role in the process of microvascular and lymphatic metastasis. Moreover, in the tumor microenvironment, ADC is usually associated with increased density of CC cells, increased nuclear-cytoplasmic ratio and reduced extracellular space. Low ADC values often indicate that tumor cells are closely arranged and have obvious atypia, which is related to higher histological grade, stronger invasiveness and poorer prognosis (8, 9). To be more specific, correlation analysis further confirmed the consistency between intratumoral and peritumoral (P < 0.05). Thus, these findings suggest that intratumoral and peritumoral radomics provide complementary insights into specific regions in the prediction of HGVI double positivity and emphasize the correlation between peritumoral radiomics and the invasivity of LVSI.

The non-linear relationship and inflection point between ADC sequence-derived intratumoral radiomics scores and the incidence of double positive HGVI may reflect the complex evolution of the tumor microenvironment at the pathological physiological and molecular cellular levels. As the malignancy of CC increases, tumor cells proliferate extensively, and cell density further rises, while ADC values continue to decrease. Additionally, the rapid growth of CC during follow-up leads to local hypoxia, which in turn induces the upregulation of molecules. This might trigger a series of cascade reactions promoting invasion and metastasis, driving tumor cells to acquire stronger migratory ability, and thus more easily invade the lymphovascular space. However, this molecular-level “qualitative change” process may be the underlying reason for the non-linear relationship between the radiomics score and double positive HGVI. Importantly, the appearance of the inflection point may indicate that the tumor microenvironment has reached a critical threshold under continuous deterioration. Therefore, the ADC sequence intratumoral radiomics score captures the continuous pathological physiological process from the increase in cell density to the deterioration of the microenvironment and ultimately the leap to the invasive phenotype. The existence of the inflection point may suggest that the tumor microenvironment has undergone irreversible deterioration at a certain node, providing an important imaging biomarker for clinical decision-making.

Importantly, SHAP analysis shows that the machine learning classifier of Xgboost contributes more to the final fusion model, which means that Xgboost can efficiently integrate multiple radiomics or imaging data, has strong feature recognition and classification capabilities, helps to accurately quantify the heterogeneity of tumor microenvironment, and may help to achieve synchronous evaluation of pathological grading and LVSI risk stratification of CC patients. Ultimately, to simultaneously evaluate the high-risk pathological features of CC with both high pathological grade and LVSI positivity, this ENMLR integrates different machine learning algorithms, multi-sequence MR radiomics of intratumoral and peritumoral 3 mm and 5mm regions, and serum biomarkers, which not only have optimal predictive value, but also good discriminative ability and stability. However, the ENMLR model may be regarded as an auxiliary decision-making and risk stratification tool rather than a diagnostic detection method. Multi-center prospective external validation is required in the future. Moreover, this model is still in the early exploration stage and has not yet received clinical application approval. Before considering clinical application, it is necessary to undergo rigorous multi-center prospective validation and obtain approval from the regulatory authorities.

Moreover, this study has several limitations. First, the sample size was relatively small and lacked external validation; future studies should incorporate multi-center cohorts to improve generalizability. Second, its retrospective design may introduce selection bias. Third, while the model simultaneously predicts HGVI double positivity of CC patients, other high-risk features such as Ki67 expression and lymph node metastasis were not integrated; subsequent work should explore multimodal approaches to jointly analyze these factors. Finally, follow-up data were unavailable, limiting prognostic validation. To be more specific, future efforts should include postoperative survival follow-up and evaluate deep learning methods for predicting HGVI double positivity in CC patients.

## 5 Conclusions

The ENMLR model—integrating ensemble machine learning, multi-sequence MR radiomics from intratumoral and peritumoral (3 mm and 5 mm) regions, and serum biomarkers—demonstrates robust predictive performance for HGVI double positivity in CC and confers enhanced clinical net benefit. RCS analysis further revealed a significant nonlinear association between ADC sequence-derived intratumoral radiomics scores and increased incidence of HGVI double positivity, with a distinct inflection point. SHAP analysis identified ADC_peritumoral_3mm and Xgboost as the most influential contributors to the fusion model. Nevertheless, the primary value of this model would likely lie in screening and risk stratification—aiding in the identification of patients who may warrant intensified surveillance or adjunctive therapy—while definitive diagnosis and treatment decisions must remain grounded in histopathological and clinical correlation. Multi-center prospective studies, postoperative survival follow-up cohorts, and translational investigations are urgently needed to validate these preliminary results and to clarify the biological relevance of the identified radiomic signatures before any clinical application can be contemplated.

## Data Availability

The original contributions presented in the study are included in the article/**Supplementary Material**. Further inquiries can be directed to the corresponding authors.
