# Supplementary material for: Interpretable ensemble learning model using intratumoral and peritumoral multi-sequence MR-radiomics predicts high-grade cervical cancer with lymphovascular space invasion
Source: Front Oncol. 2026 Sep 9;16:1827751. doi: 10.3389/fonc.2026.1827751 (PMC13597462; doi:10.3389/fonc.2026.1827751)
Supplement: Supplementary file 1 [file DataSheet1.pdf]

# *Supplementary Material*

## 1.Supplementary Tables

**Supplementary Table 1：**

The Optimal cut-off values for predicting HGVI dual-positive in CC using ROC curve analysis.

| **Biomarkers** | **Serum tumor biomarkers** | **Optimal cutoff value** |
|---|---|---|
| WBC | White blood cell count | 7.38 |
| Neu | Neutrophilicgranulocyte count | 4.84 |
| Lym | Lymphocyte count | 1.77 |
| MO | Monocyte count | 0.51 |
| PLT | Platelet count | 237.00 |
| ALT | Alanine aminotransferase | 29.00 |
| AST | Aspartate aminotransferase | 20.00 |
| ALP | Alkaline phosphatase | 61.00 |
| GGT | Gamma-glutamyl transferase | 14.40 |
| CA199 | Carbohydrate antigen 199 | 25.41 |
| CEA | Carcinoembryonic antigen | 42.30 |
| CA125 | Carbohydrate antigen 125 | 5.79 |

Abbreviations: HGVI, High pathological grading and lymphovascular space invasion; ROC, the receiver operating characteristic curve; CC, Cervical cancer.

**Supplementary Table 2:**

**Scan Parameters of Siemens(3.0T)**

| **Sequences** | **TR (ms)** | **TE (ms)** | **FOV ($mm^2$)** | **Layer thickness (mm)** | **Matrix** | **BW (Hz)** |
|---|---|---|---|---|---|---|
| $T_1$-weighted imaging VIBE | 3.97 | 1.23 | 380×274 | 3 | 146×288 | 1020 |
| $T_2$-weighted imaging | 5560 | 88 | 260×260 | 3 | 272×320 | 279 |
| DWI # | 5100 | 79 | 340×244 | 4 | 78×128 | 2170 |

Abbreviations: TR, Repetition time; TE, Echo time; FOV, Field of view; BW, Bandwidth; $T_1$WI, $T_1$-weighted imaging; $T_2$WI, $T_2$-weighted imaging; DWI, Diffusion-weighted Imaging; VIBE, Volumetric interpolated breath-hold examination.

# DWI was performed with b value taken as 0, and 800 s/$mm^2$.

**Supplementary Table 3:**

The optimal cut-off values for predicting HGVI dual-positive of serum biomarkers in the training set and the test set.

| Variables | Training set | | | Test set | | |
|---|---|---|---|---|---|---|
| | Non-dual-positive group(N=105) | Dual-positive group(N=64) | P values | Non-dual-positive group(N=50) | Dual-positive group(N=23) | P values |
| WBC≥7.38: | | | 0.085 | | | 0.560 |
| NO | 71 (67.6%) | 34 (53.1%) | | 23 (46.0%) | 13 (56.5%) | |
| YES | 34 (32.4%) | 30 (46.9%) | | 27 (54.0%) | 10 (43.5%) | |
| Neu≥4.84: | | | 0.052 | | | 1 |
| NO | 65 (61.9%) | 29 (45.3%) | | 23 (46.0%) | 11 (47.8%) | |
| YES | 40 (38.1%) | 35 (54.7%) | | 27 (54.0%) | 12 (52.2%) | |
| Lym≤1.77: | | | 0.132 | | | 0.974 |
| NO | 32 (30.5%) | 12 (18.8%) | | 17 (34.0%) | 7 (30.4%) | |
| YES | 73 (69.5%) | 52 (81.2%) | | 33 (66.0%) | 16 (69.6%) | |
| MO≥0.51: | | | 0.053 | | | 0.780 |
| NO | 80 (76.2%) | 39 (60.9%) | | 36 (72.0%) | 18 (78.3%) | |
| YES | 25 (23.8%) | 25 (39.1%) | | 14 (28.0%) | 5 (21.7%) | |
| PLT≥237.0: | | | 0.375 | | | 0.164 |
| NO | 48 (45.7%) | 24 (37.5%) | | 18 (36.0%) | 13 (56.5%) | |
| YES | 57 (54.3%) | 40 (62.5%) | | 32 (64.0%) | 10 (43.5%) | |
| ALT≤29.0: | | | 0.038 | | | 0.196 |
| NO | 15 (14.3%) | 2 (3.1%) | | 3 (6.00%) | 4 (17.4%) | |
| YES | 90 (85.7%) | 62 (96.9%) | | 47 (94.0%) | 19 (82.6%) | |
| AST≥20.0: | | | 0.277 | | | 0.287 |
| NO | 53 (50.5%) | 26 (40.6%) | | 30 (60.0%) | 10 (43.5%) | |
| YES | 52 (49.5%) | 38 (59.4%) | | 20 (40.0%) | 13 (56.5%) | |
| ALP≤61.0: | | | 0.121 | | | 0.129 |
| NO | 94 (89.5%) | 51 (79.7%) | | 46 (92.0%) | 18 (78.3%) | |
| YES | 11 (10.5%) | 13 (20.3%) | | 4 (8.00%) | 5 (21.7%) | |
| GGT≤14.4: | | | 0.123 | | | 0.498 |
| NO | 90 (85.7%) | 48 (75.0%) | | 38 (76.0%) | 15 (65.2%) | |
| YES | 15 (14.3%) | 16 (25.0%) | | 12 (24.0%) | 8 (34.8%) | |
| CA199≥25.41: | | | 0.056 | | | 0.497 |
| NO | 74 (70.5%) | 35 (54.7%) | | 36 (72.0%) | 14 (60.9%) | |
| YES | 31 (29.5%) | 29 (45.3%) | | 14 (28.0%) | 9 (39.1%) | |
| CA125≥42.3: | | | <0.001 | | | 0.002 |
| NO | 89 (84.8%) | 36 (56.2%) | | 41 (82.0%) | 10 (43.5%) | |
| YES | 16 (15.2%) | 28 (43.8%) | | 9 (18.0%) | 13 (56.5%) | |
| CEA≥5.79: | | | 0.120 | | | 0.119 |
| NO | 65 (61.9%) | 31 (48.4%) | | 33 (66.0%) | 10 (43.5%) | |
| YES | 40 (38.1%) | 33 (51.6%) | | 17 (34.0%) | 13 (56.5%) | |

Abbreviations: HGVI, High pathological grading and lymphovascular space invasion.

**Supplementary Table 4:**

Feature screening of clinical biomarkers using LASSO regression and Boruta algorithm

| **Variables** | **Boruta algorithm** | **LASSO regression** |
|---|---|---|
| Feature name | "Lym" "ALT" "AST" "ALP" "GGT" "CA125" | "ALT" "ALP" "GGT" "CA125" |
| Common subset | "ALT" "ALP" "GGT" "CA125" | |

Abbreviations: LASSO, least absolute shrinkage and selection operator.

**Supplementary Table 5:**

The specific number of features in the radiomics-feature filtering process.

| **Radiomics feature screening** | **Feature number** | **ICC≥0.8; Normalize with Z-score** | **T-test; Mann-Whitney *U*; Spearman's rank correlation > 0.9; mRMR** | **Top 10 radiomics features based on SHAP algorithm** | **Optimal number based on Boruta algorithm** |
|---|---|---|---|---|---|
| **ADC** | | | | | |
| Intratumor | 1130 | 987 | 232 | 10 | 4 |
| Peritumoral 3mm | 1130 | 987 | 217 | 10 | 3 |
| Peritumoral 5mm | 1130 | 987 | 217 | 10 | 4 |
| **$T_1$WI** | | | | | |
| Intratumor | 1130 | 987 | 277 | 10 | 4 |
| Peritumoral 3mm | 1130 | 987 | 217 | 10 | 4 |
| Peritumoral 5mm | 1130 | 987 | 254 | 10 | 1 |
| **$T_2$WI** | | | | | |
| Intratumor | 1130 | 987 | 204 | 10 | 5 |
| Peritumoral 3mm | 1130 | 987 | 204 | 10 | 5 |
| Peritumoral 5mm | 1130 | 987 | 195 | 10 | 4 |

Abbreviations: LASSO, least absolute shrinkage and selection operator; ICC, Intraclass correlation coefficient; mRMR, Maximum Relevance Minimum Redundancy; ADC, apparent diffusion coefficient; $T_1$WI, T1-weighted imaging; $T_2$WI, T2-weighted imaging.

**Supplementary Table 6:**

The detailed calculation and construction process of ADC (**a**), $T_1$WI (**b**), $T_2$WI (**c**), perStacking and intraStacking (**d**).

ADC (**a**)**:**

| Peritumoral 3mm | Feature Name | Coefficient values |
|---|---|---|
| **ADC 3mmRS** | Intercept | -2.061175 |
| **A1** | "wavelet.HLL_firstorder_Skewness" | 1.829895 |
| **A2** | "wavelet.LLL_firstorder_Minimum" | 1.672516 |
| **A3** | "log.sigma.5.0.mm.3D_glszm_ZoneVariance" | -0.0359219 |
| **ADC 3mmRS**=-2.061175+**A(1-3)***Coefficient values | | |

| Peritumoral 5mm | Feature Name | Coefficient values |
|---|---|---|
| **ADC 5mmRS** | Intercept | -1.675406 |
| **B1** | "original_shape_Elongation" | 1.54578 |
| **B2** | "wavelet.HLL_firstorder_Skewness" | 0.1710976 |
| **B3** | "wavelet.LLL_glszm_ZoneVariance" | 0.1457838 |
| **B4** | "wavelet.LLL_glszm_GrayLevelVariance" | 0.4154888 |
| **ADC 5mmRS**=-1.675406+**B(1-4)***Coefficient values | | |

| Intratumor | Feature Name | Coefficient values |
|---|---|---|
| **ADCRS** | Intercept | -3.758476 |
| **C1** | "wavelet.LLH_gldm_DependenceNonUniformityNormalized" | 5.693059 |
| **C2** | "wavelet.LHH_firstorder_Kurtosis" | 0.7552751 |
| **C3** | "wavelet.LHL_firstorder_Kurtosis" | 1.122016 |
| **C4** | "wavelet.LLH_gldm_DependenceVariance" | 2.931782 |
| **ADCRS**=-3.758476+**C(1-4)***Coefficient values | | |
| **perADCRS**=0.539389+1.038418***ADC 3mmRS**+1.049132***ADC 5mmRS** | | |

$T_1$WI (**b**):

| Peritumoral 3mm | Feature Name | Coefficient values |
|---|---|---|
| **T1WI 3mmRS** | Intercept | -1.675406 |
| **D1** | "original_shape_Elongation" | 1.54578 |
| **D2** | "wavelet.HLL_firstorder_Skewness" | 0.1710976 |
| **D3** | "wavelet.LLL_glszm_ZoneVariance" | 0.1457838 |
| **D4** | "wavelet.LLL_glszm_GrayLevelVariance" | 0.4154888 |
| **T1WI 3mmRS**=-1.675406+**D(1-4)***Coefficient values | | |

| Peritumoral 5mm | Feature Name | Coefficient values |
|---|---|---|
| **T1WI 5mmRS** | Intercept | -0.3554309 |

| | | |
|---|---|---|
| **E1** | "original_glszm_SmallAreaLowGrayLevelEmphasis" | -1.328418 |
| **T1WI 5mmRS**=-0.3554309+**E(1)***Coefficient values | | |
| Intratumor | Feature Name | Coefficient values |
| **T1WIRS** | Intercept | -2.270786 |
| **F1** | "log.sigma.5.0.mm.3D_glrlm_LongRunHighGrayLevelEmphasis" | 2.15269 |
| **F2** | "wavelet.HLL_firstorder_Mean" | 5.721029 |
| **F3** | "wavelet.LLH_ngtdm_Complexity" | -1.642048 |
| **F4** | "wavelet.LLH_firstorder_Skewness" | -1.250798 |
| **T1WIRS**=-2.270786+**F(1-4)***Coefficient values | | |
| **perT1WIRS**=0.70962479+1.025969***T1WI 3mmRS**+1.156675***T1WI 5mmRS** | | |

$T_2$WI (**c**):

| Peritumoral 3mm | Feature Name | Coefficient values |
|---|---|---|
| **T2WI 3mmRS** | Intercept | -0.2520377 |
| **G1** | "wavelet.LHL_glcm_MCC" | 0.8847483 |
| **G2** | "wavelet.HLH_gldm_SmallDependenceHighGrayLevelEmphasis" | 0.5746493 |
| **G3** | "wavelet.HHL_gldm_DependenceVariance" | 1.421006 |
| **G4** | "log.sigma.3.0.mm.3D_glszm_SmallAreaEmphasis" | -1.976772 |
| **G5** | "wavelet.LHH_glszm_LowGrayLevelZoneEmphasis" | -1.376306 |
| **T2WI 3mmRS**=-0.2520377+**G(1-5)***Coefficient values | | |
| Peritumoral 5mm | Feature Name | Coefficient values |
| **T2WI 5mmRS** | Intercept | -4.317005 |
| **H1** | "wavelet.LHL_glcm_MCC" | +1.022211 |
| **H2** | "original_glcm_Idmn" | 3.031146 |
| **H3** | "wavelet.LHH_glcm_MCC" | 5.140299 |
| **H4** | "wavelet.HHH_glrlm_ShortRunHighGrayLevelEmphasis" | -0.4431784 |
| **T2WI 5mmRS**=-4.317005+**H(1-4)***Coefficient values | | |
| Intratumor | Feature Name | Coefficient values |
| T2WIRS | Intercept | -6.051664 |
| **I1** | "wavelet.HLH_glszm_ZoneVariance" | 0.5492122 |
| **I2** | "wavelet.LHH_glcm_MCC" | 2.385905 |
| **I3** | "wavelet.HHL_firstorder_Mean" | 4.441582 |
| **I4** | "wavelet.LHL_glcm_Idn" | 1.761324 |
| **I5** | "original_gldm_DependenceVariance" | 0.1654258 |
| **T2WIRS**=-6.051664+**I(1-5)***Coefficient values | | |

| |
|---|
| **perT2WIRS**=0.295535+0.7771175***T2WI 3mmRS**+0.8463878***T2WI 5mmRS** |

perStacking and intraStacking (**d**):

**perStacking**=0.3854113

+1.006357***perADCRS**

-0.2002035***perT1WIRS**

+1.005815***perT2WIRS**

**intraStacking**=1.136919

+0.4301028***ADCWIRS**

+0.8733278***T1WIRS**

+0.8822116***T2WIRS**

Abbreviations: ADC, apparent diffusion coefficient; T1WI, T1-weighted imaging; T2WI, T2-weighted imaging; RS, the radiomics score of radiomics features; perStacking, the stacking radiomics scores based on peritumoral 3mm and peritumoral 5mm radiomics features of ADC, T1WI and T2WI; intraStacking, the stacking radiomics scores based on intratumoral radiomics features of ADC, T1WI and T2WI.

## 2. Supplementary Figures

**Supplementary Figure 1:**

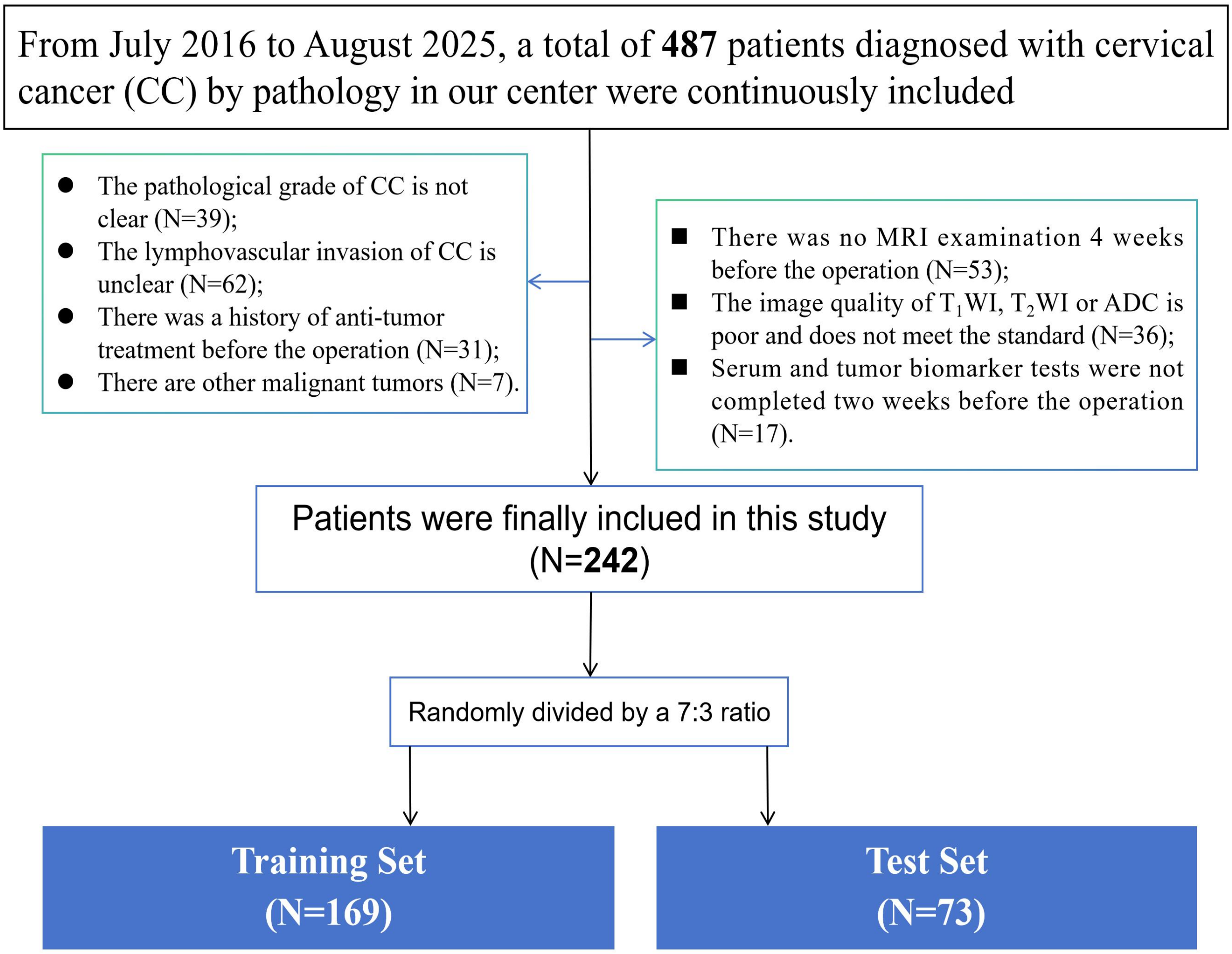


The flowchart of patient screening.

**Supplementary Figure 2:**

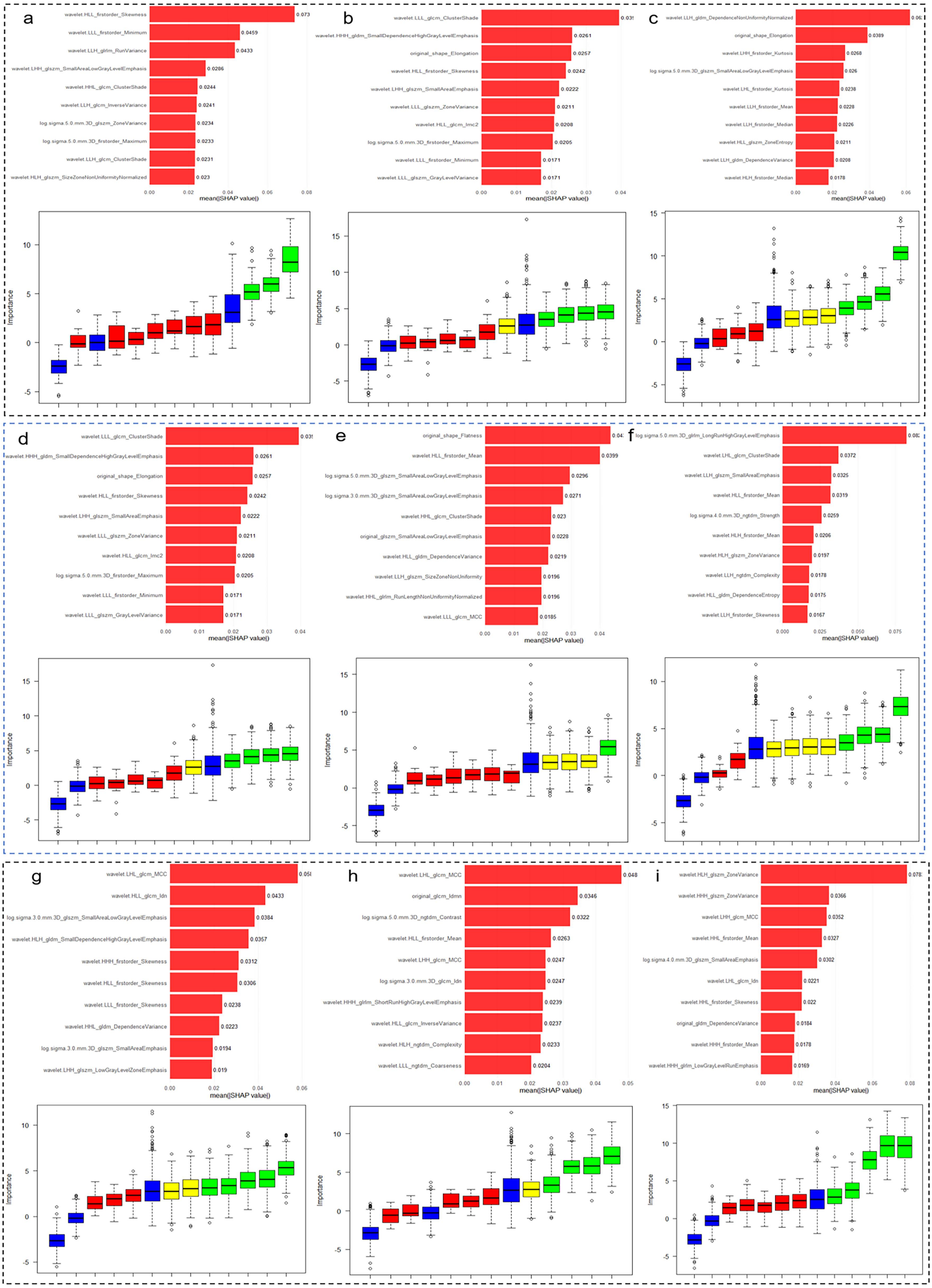

Radiomics feature screening using the SHAP and Boruta algorithm. The top ten most contributive features and the final modeling radiomics features are retained through the SHAP and Boruta algorithm for ADC sequence (**a**: peritumoral 3 mm; **b**: peritumoral 5 mm; **c**: intratumor), $T_1$WI sequence (**d**: peritumoral 3 mm; **e**: peritumoral 5 mm; **f**: intratumor), and $T_2$WI sequence (**g**: peritumoral 3 mm; **h**: peritumoral 5 mm; **i**: intratumor).

Abbreviations: SHAP, the SHapley Additive exPlanations algorithm; ADC, apparent diffusion coefficient; $T_1$WI, T1-weighted imaging; $T_2$WI, T2-weighted imaging.

**Supplementary Figure 3:**

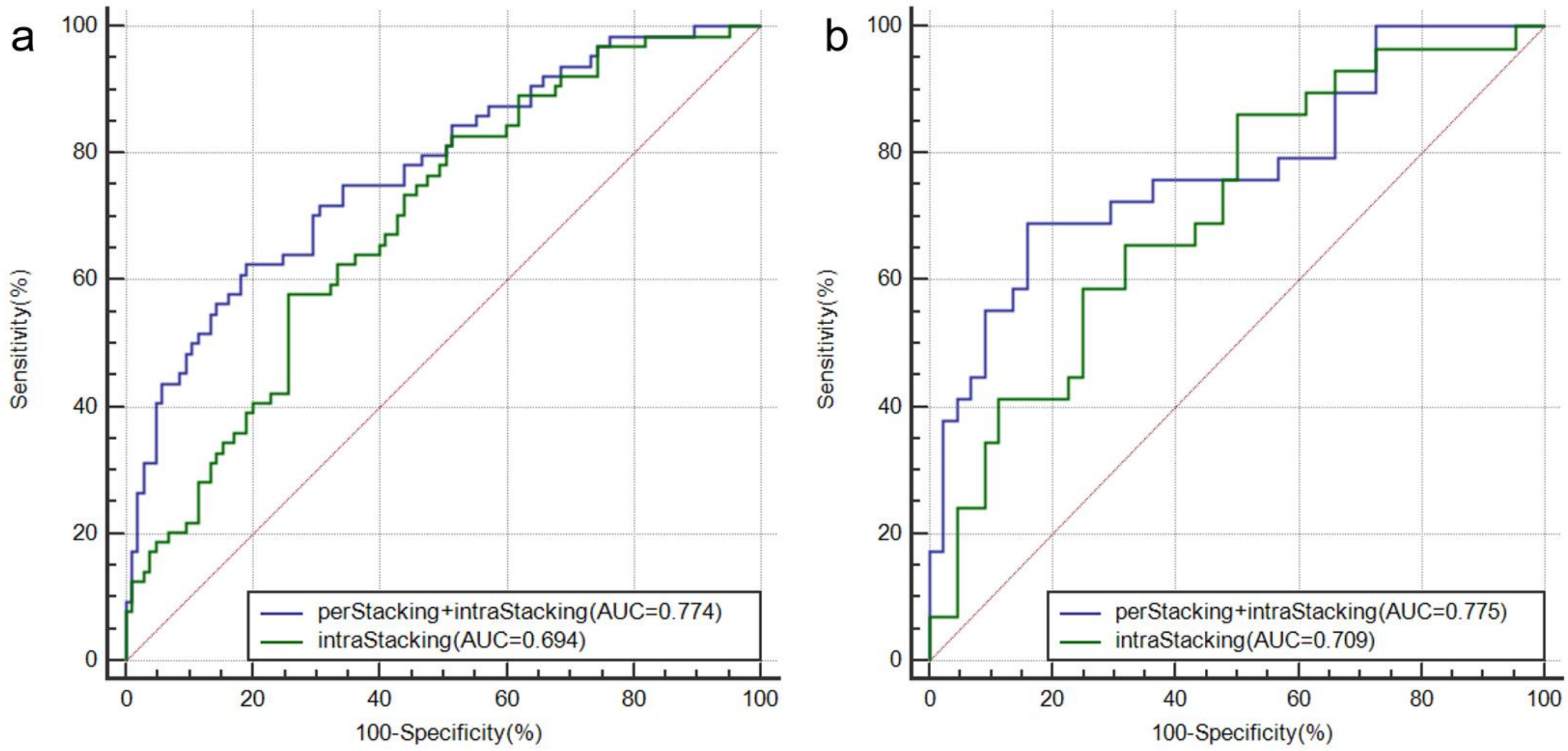


The peritumoral radiomics has supplementary value for intratumoral radiomics in predicting HGVI double positive in both the training set (**a**; AUC: increased from 0.694 to 0.774) and the test set (**b**; AUC: increased from 0.709 to 0.775).

## Supplementary Figure 4:

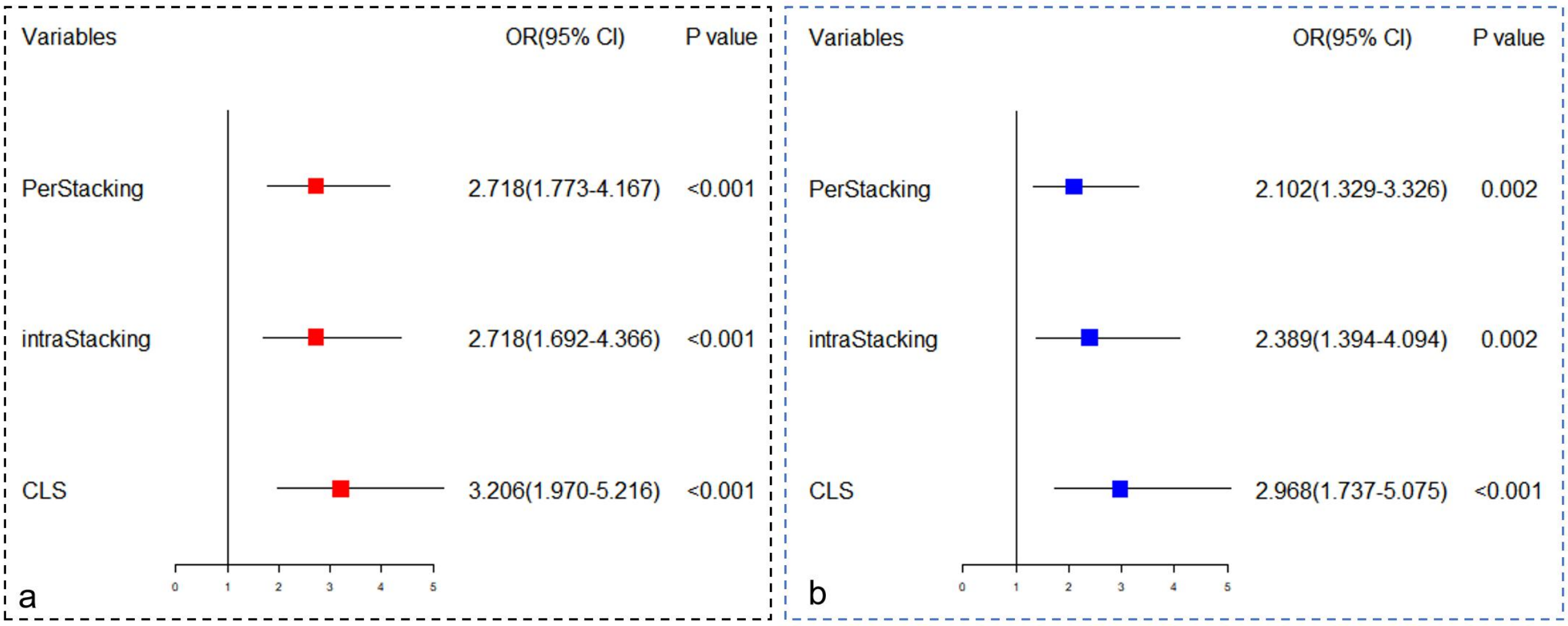


The forest diagram shows the process of the univariate logistic regression analysis (**a**) and multivariate logistic regression analysis (**b**).

## Supplementary Figure 5:

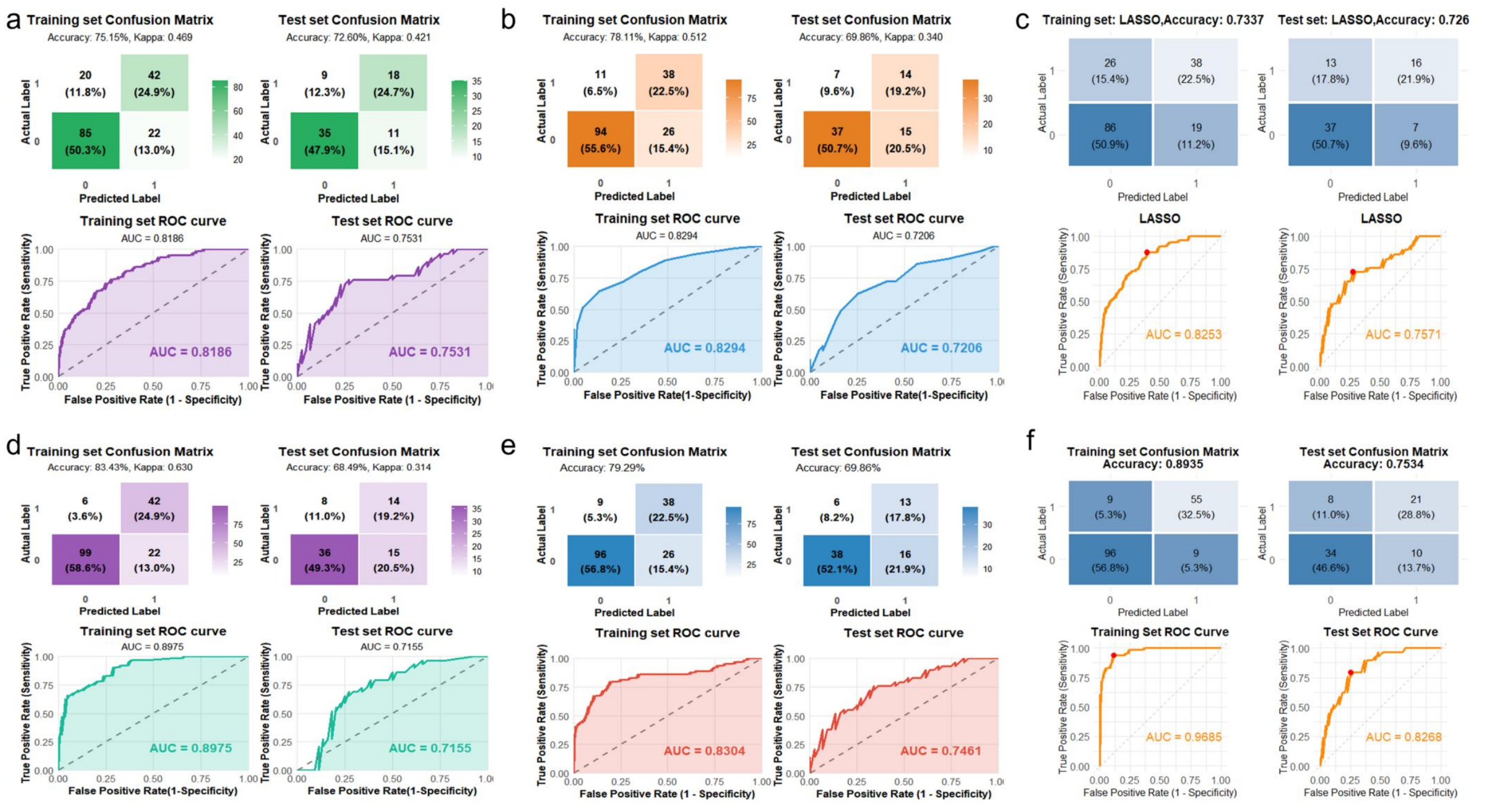


ROC curve and confusion matrix of Bayes (**a**), KNN (**b**), LR (**c**), NNet (**d**), SVM (**e**), Xgboost (**f**) for predicting HGVI double positivity in CC.

Abbreviations: ROC, receiver operating characteristic curve; KNN, k-nearest neighbor; LR, logistic regression; NNet, neural network; SVM, support vector machine; Xgboost, extreme gradient

boosting classifiers; HGVI, High pathological grading and lymphovascular space invasion; CC, Cervical cancer.

**Supplementary Figure 6:**

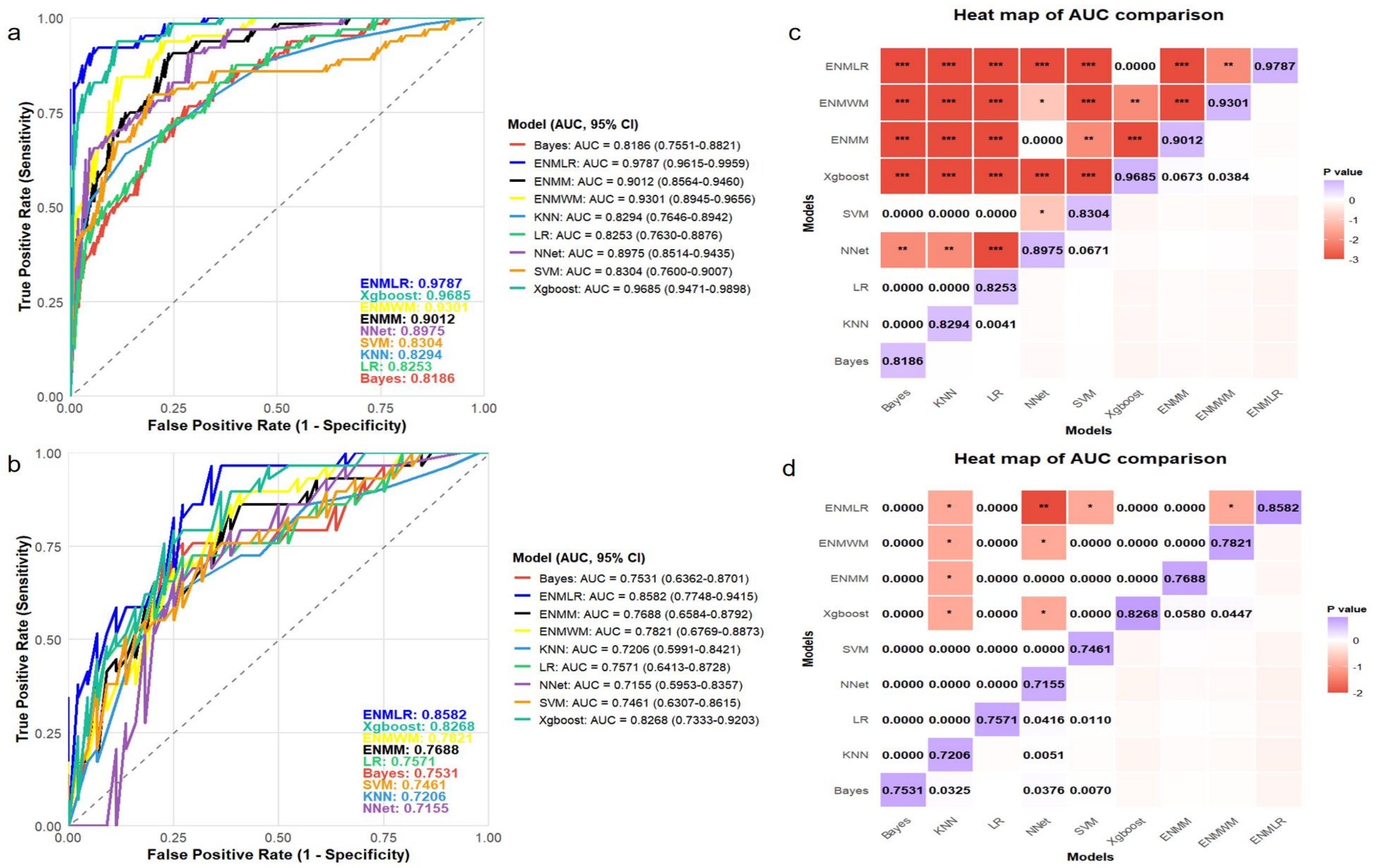


ROC curve analysis (**a**, **b**) and heat map analysis (**c**, **d**) of 9 models. The ROC curve analysis and heat map analysis indicated that ENMLR obtained more predictive performance than the other models in the training set (**a**, **c**) and test set (**b**, **d**).

**Supplementary Figure 7:**

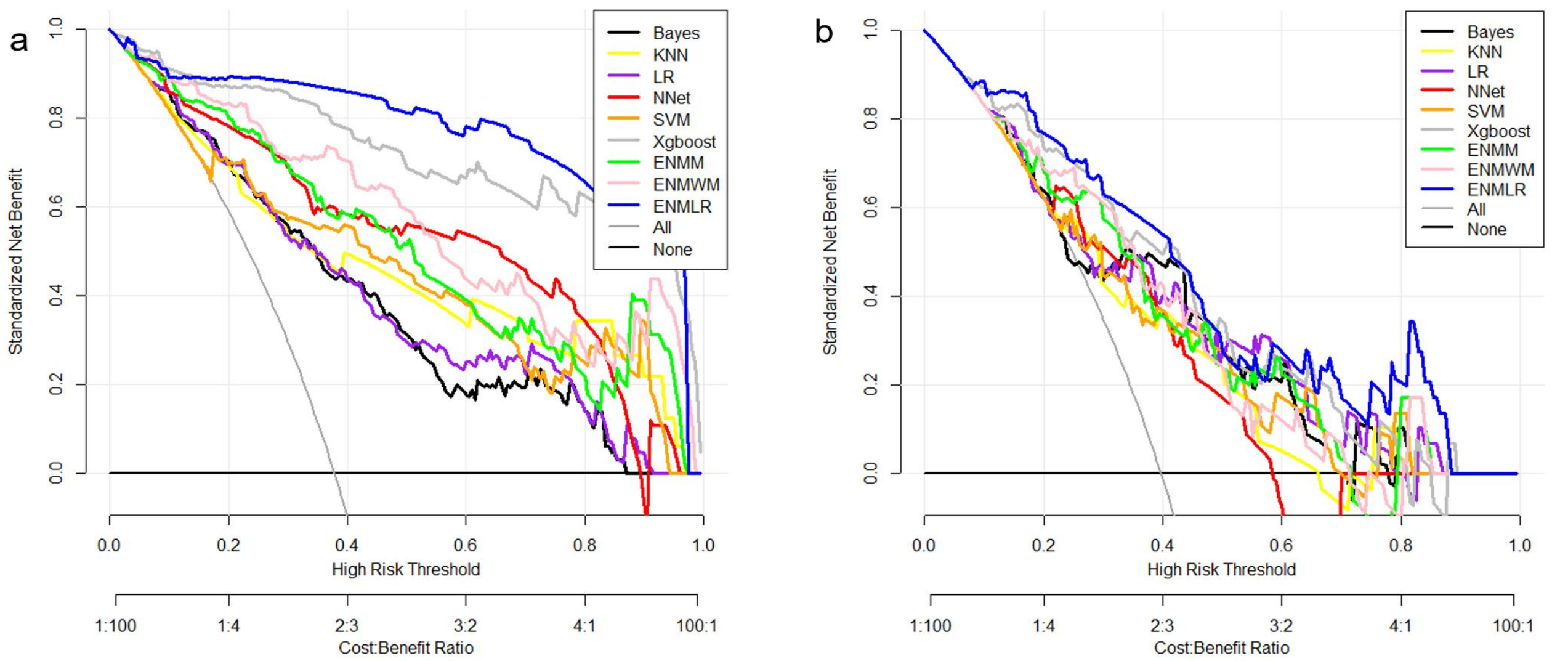


The DCA indicates that ENMLR achieved more clinical net benefits to predict HGVI double positivity of CC than the other models in the training set (**a**) and test set (**b**).
